# Multicenter Study Demonstrates Standardization Requirements for Mold Identification by MALDI-TOF MS

**DOI:** 10.3389/fmicb.2019.02098

**Published:** 2019-09-20

**Authors:** Anna F. Lau, Robert C. Walchak, Heather B. Miller, E. Susan Slechta, Kamal Kamboj, Katherine Riebe, Amy E. Robertson, Jeremy J. Gilbreath, Kaitlin F. Mitchell, Meghan A. Wallace, Alexandra L. Bryson, Joan-Miquel Balada-Llasat, Amanda Bulman, Blake W. Buchan, Carey-Ann D. Burnham, Susan Butler-Wu, Uma Desai, Christopher D. Doern, Kimberly E. Hanson, Christina M. Henderson, Markus Kostrzewa, Nathan A. Ledeboer, Thomas Maier, Preeti Pancholi, Audrey N. Schuetz, Gongyi Shi, Nancy L. Wengenack, Sean X. Zhang, Adrian M. Zelazny, Karen M. Frank

**Affiliations:** ^1^Department of Laboratory Medicine, Clinical Center, National Institutes of Health, Bethesda, MD, United States; ^2^Division of Clinical Microbiology, Mayo Clinic, Rochester, MN, United States; ^3^Department of Pathology, Johns Hopkins University School of Medicine, Baltimore, MD, United States; ^4^ARUP Institute for Clinical and Experimental Pathology, Salt Lake City, UT, United States; ^5^Department of Pathology, The Ohio State University Wexner Medical Center, Columbus, OH, United States; ^6^Department of Pathology, Medical College of Wisconsin, Milwaukee, WI, United States; ^7^Clinical Microbiology Laboratory, Weill Cornell Medical Center/New York Presbyterian Hospital, New York, NY, United States; ^8^Department of Laboratory Medicine, University of Washington, Seattle, WA, United States; ^9^Department of Pathology and Immunology, Washington University School of Medicine, St. Louis, MO, United States; ^10^Department of Pathology, Virginia Commonwealth University School of Medicine, Richmond, VA, United States; ^11^Bruker Daltonics, Inc., Billerica, MA, United States; ^12^Department of Pathology, Division of Clinical Microbiology, The University of Utah, Salt Lake City, UT, United States

**Keywords:** mold, filamentous fungi, MALDI-TOF MS, rapid, identification

## Abstract

**Objectives:**

Rapid and accurate mold identification is critical for guiding therapy for mold infections. MALDI-TOF MS has been widely adopted for bacterial and yeast identification; however, few clinical laboratories have applied this technology for routine mold identification due to limited database availability and lack of standardized processes. Here, we evaluated the versatility of the NIH Mold Database in a multicenter evaluation.

**Methods:**

The NIH Mold Database was evaluated by eight US academic centers using a solid media extraction method and a challenge set of 80 clinical mold isolates. Multiple instrument parameters important for spectra optimization were evaluated, leading to the development of two specialized acquisition programs (NIH method and the Alternate-B method).

**Results:**

A wide range in performance (33–77%) was initially observed across the eight centers when routine spectral acquisition parameters were applied. Use of the NIH or the Alternate-B specialized acquisition programs, which are different than those used routinely for bacterial and yeast spectral acquisition (MBT_AutoX), in combination with optimized instrument maintenance, improved performance, illustrating that acquisition parameters may be one of the key limiting variable in achieving successful performance.

**Conclusion:**

Successful mold identification using the NIH Database for MALDI-TOF MS on Biotyper systems was demonstrated across multiple institutions for the first time following identification of critical program parameters combined with instrument optimization. This significantly advances our potential to implement MALDI-TOF MS for mold identification across many institutions. Because instrument variability is inevitable, development of an instrument performance standard specific for mold spectral acquisition is suggested to improve reproducibility across instruments.

## Introduction

Rapid bacterial and yeast identification by matrix-assisted laser desorption ionization-time of flight mass spectrometry (MALDI-TOF MS) has revolutionized microbiology laboratory practices (Tan et al., 2012; Huang et al., 2013; Lacroix et al., 2014). Studies evaluating MALDI-TOF MS for mold identification have been published, but only a few clinical laboratories have applied this technology in routine practice. Limiting factors have included (1) poor performance of manufacturer’s databases leading to a heavy dependency on laboratory-developed databases (including online databases such as the Mass Spectrometry Identification Platform (MSI) (Normand et al., 2017) and MicrobeNet from the Centers for Disease Control and Prevention (CDC) (Center for Disease Control and Prevention [CDCP], 2019); (2) lack of standardized processing methods (Alanio et al., 2011; Cassagne et al., 2011; Theel et al., 2011; Alshawa et al., 2012; De Carolis et al., 2012; Del Chierico et al., 2012; Schrodl et al., 2012; de Respinis et al., 2013; Lau et al., 2013; L’Ollivier et al., 2013; Nenoff et al., 2013; Normand et al., 2013; Barker et al., 2014; Becker et al., 2014; Gautier et al., 2014; Ranque et al., 2014; Sitterle et al., 2014; Dolatabadi et al., 2015; Triest et al., 2015; Sleiman et al., 2016); and (3) the absence of an FDA-cleared mold database until recent release of the VITEK MS 3.0 (bioMerieux, Inc., Durham, NC, United States) which includes representation of 47 filamentous fungi (United States Food and Drug Administration, 2017). Many clinical laboratories worldwide, however, utilize either the Bruker MALDI Biotyper (MBT) system for which the Filamentous Fungal Database has not been cleared by the FDA, or the older VITEK MS 2.0 which lacks the mold database due to the time needed for test system reverification and/or software compatibility restraints.

Since 2012, the National Institutes of Health (NIH) Clinical Center has applied MALDI-TOF MS for the routine clinical identification of molds for patient care directly from solid media using the NIH Mold Database (available publicly since 2013) to supplement that of the Bruker MBT system (Lau et al., 2013). To investigate the broader utility of the NIH Mold Database beyond our institution, a multicenter study including eight US academic institutions was conducted. Unlike multicenter bacterial and yeast studies for which excellent inter- and intra-laboratory reproducibility has been demonstrated (Westblade et al., 2015; Wilson et al., 2017), this study has highlighted significant performance variability between instruments regardless of acquisition parameters and demonstrated that development of a specific mold standard is required for successful use of MALDI-TOF MS for mold identification.

## Materials and Methods

### Isolates

Eighty clinical isolates (10 from each of the eight original participating centers – National Institutes of Health, Bethesda, MD; Mayo Clinic, Rochester, MN; The Ohio State University Wexner Medical Center, Columbus, OH; ARUP Institute for Clinical and Experimental Pathology, Salt Lake City, UT; Medical College of Wisconsin, Milwaukee, WI; University of Washington, Seattle, WA; Johns Hopkins University School of Medicine, Baltimore, MD; and Weill Cornell Medical Center/New York Presbyterian Hospital, New York, NY, United States) representing a diverse group of common clinical molds and less common organisms were studied (Table 1). An additional two centers (Washington University and Virginia Commonwealth University) assisted with testing at the final stages of the study. All 80 isolates were shipped to the NIH, blinded, subcultured onto Sabouraud Dextrose Agar (SDA) slants, and then redistributed to each of the centers. Upon receipt, isolates were subcultured onto SDA prior to protein extraction performed at each of the individual laboratories. To effectively challenge the specificity of the NIH Mold Database, some isolates that were not well-represented in the database were purposefully selected. Gold standard identification for each isolate included macroscopic and microscopic morphologies with confirmation by Sanger sequencing of the ITS1-2 (White et al., 1990), when necessary. Species-level identification was not possible for all isolates as the ITS region was unable to provide sufficient discriminatory power following MM18 guidelines provided by the Clinical Laboratory Standards Institute (Clinical and Laboratory Standards Institute [CLSI], 2008).

**TABLE 1:** List of mold isolates used in study (*n* = 80).

**Hyaline (*n* = 36)**	**Dematiaceous (*n* = 22)**	**Mucorales (*n* = 9)**	**Dermatophytes (*n* = 7)**	**Dimorphic fungi (*n* = 6)**
*Acremonium* spp. (2) *Aspergillus fumigatus* (3) *Aspergillus glaucus* group (1) *Aspergillus niger* (1) *Aspergillus sydowii* (1)^∗^ *Aspergillus terreus* (2)^∗^ *Aspergillus ustus* (1)^∗^ *Aspergillus viridinutans* (1) *Beauveria bassiana* (2) *Eutypella* spp. (1) *Fusarium oxysporum* (1) *Fusarium solani* (1)^∗^ *Fusarium* spp. (2) *Fusarium verticillioides* (1) *Hormographiella* spp. (1) *Metarhizium* spp. (1) *Myrmecridium* spp. (1) *Neosartorya hiratsukae* (1) *Neosartorya udagawae* (1) *Paecilomyces saturatum* (1) *Paecilomyces variotii* (1) *Penicillium* spp. (2) *Purpureocillium lilacinum* (2)^∗^ *Rasamsonia argillacea* (3)^∗^ *Scopulariopsis brevicaulis* (1) *Trichoderma* spp. (1)	*Alternaria* spp. (2) *Aureobasidium pullulans* (1) *Chaetomium* spp. (2) *Cladophialophora bantiana* (1) *Cladosporium* spp. (1) *Epicoccum* spp. (1) *Exophiala dermatitidis* (2)^∗^ *Exserohilum rostratum* (1)^∗^ *Lomentospora prolificans* (2) *Ochroconis* spp. (2)^∗^ *Phaeoacremonium* spp. (1) *Phialophora* spp. (1) *Phoma* spp. (2) *Scedosporium apiospermum* (1)^∗^ *Scedosporium dehoogii* (1) *Scytalidium* spp. (1)	*Cunninghamella elegans* (3) *Lichtheimia corymbifera* (2) *Mucor* spp. (1)^∗^ *Rhizopus oryzae* (1) *Syncephalastrum* spp. (2)^∗^	*Microsporum canis* (3) *Trichophyton rubrum* (2) *Trichophyton* spp. (2)^∗^	*Blastomyces dermatitidis* (2) *Histoplasma capsulatum* (2) *Sporothrix schenckii* (2)

*^∗^Subset of representative strains used in later analyses for comparison of spectral acquisition methods (*n* = 17; NIH mold study strains 7, 11, 18, 20, 22–24, 26, 28–29, 45, 64, 66, 67, 71, 76, and *A. ustus* CBS 261.67T control strain).*

### Protein Extraction and MALDI-TOF MS

Extraction was performed using the NIH solid media extraction method as described previously (Lau et al., 2013) and in this video^1^. Spectra were collected on MicroFlex LT instruments (Bruker Daltonics, Inc.). The same versions of the NIH database and Biotyper MBT (including the Filamentous Fungal Database) were applied for spectral analysis in all centers. Programing instructions for the NIH and Alternate-B spectral acquisition methods are provided in Supplementary Figures 1, 2.

### Study Design

Instrument model, software, database types and versions, isolates, and identification criteria were identical across the testing centers. Operators at each center varied in mass spectrometry skill level and experience, reflecting the diversity observed in clinical laboratories. Three major investigative arms were conducted sequentially – (1) evaluation of database performance using routine spectral acquisition programs set at each institution; (2) a pilot evaluation of database performance following optimization of instrument settings; and (3) evaluation of database performance using different spectral acquisition methods (NIH and Alternate-B). After analysis at each institution, extracts were frozen and transferred to the NIH for further testing. Statistical analyses were performed using Fisher’s exact test and two-tailed *p*-value^2^.

## Results

### Wide Variation in Performance Between Centers Using Routine Spectral Acquisition Programs

Following organism distribution with a set of written extraction procedural instructions to each center, species-level identification (score ≥ 2.0) ranged from 32 to 77% across the eight original centers; Center 1 (NIH instrument) performed significantly better than others (Table 2). Without compromising accuracy, identification improved to 53–85% if the score acceptability threshold was lowered to ≥ 1.7. Nine (11%) isolates (*Acremonium* spp., *Aureobasidium pullulans*, *Chaetomium* spp., *Metarhizium* spp., *Penicillium* spp., *Phialophora* spp., *Phoma* spp., *Sporothrix schenckii*, and *Syncephalastrum* spp.) failed to identify by the NIH database across all eight centers consistent with the database containing limited strain representation of some molds (maximum performance capacity of 89% from this set of 80 isolates). We purposefully created a challenge set beyond common and highly represented molds. No misidentifications were observed with the NIH Mold Database; a few misidentifications were noted with the Bruker MBT Database (Table 2).

**TABLE 2:** Initial Comparison of Mold Identification Performance at Eight Different Institutions (*n* = 80 isolates).

**Center**	**NIH Mold Database (% accuracy)**			**Bruker MBT Database^#^ (including Filamentous Fungal Database; % accuracy)**			***p*-Value (NIH vs. Bruker MBT database)**		***p*-Value (Center 1 vs. Centers 2–8; NIH database)**	
	**≥2.0**	**≥1.7**	**Mis identified**	**≥2.0**	**≥1.7**	**Misidentified**	**≥2.0**	**≥1.7**	**≥2.0**	**≥1.7**
1	77	85	0	41	60	4	<0.0001	0.0006	NA	NA
2A^∗^	33	53	0	3	19	1	<0.0001	<0.0001	<0.0001	<0.0001
3	46	68	0	40	50	4	0.5	0.04	<0.0001	0.02
4	51	71	0	6	19	1	<0.0001	<0.0001	0.0008	0.05
5	53	65	0	33	49	0	0.02	0.05	0.002	0.006
6	54	74	0	5	18	1	<0.0001	<0.0001	0.003	0.1
7	32	56	0	32	54	5	1.000	0.7	<0.0001	0.0006
8	33	61	0	11	29	3	0.001	0.0002	<0.0001	0.001

*^#^Database developed for liquid fungal cultures. ^∧^*Aspergillus sydowii*, *Aspergillus viridinutans*, *Fusarium verticillioides*, and *S. schenckii* were misidentified as *Aspergillus versicolor*, *Aspergillus fumigatus*, *Fusarium proliferatum*, and *Candida chodatii*, respectively. ^∗^Center 2 has three instruments (2A-C). Testing for this experiment was performed on instrument 2A only. Instruments 2B and 2C were tested in later phases of the study and are discussed in other Figures.*

To determine if performance variation may be due to misinterpretation of initial instructions, study participants were asked to review a video of the protein extraction procedure after which fresh subcultures of the 80 isolates were re-extracted at each site. Overall performance, however, did not improve with Center 1 continuing to perform significantly better than the other nine instruments at Centers 2–8 (Table 3). Frozen protein extracts from each site were sent to the NIH to assess the quality of the protein extraction technique. Despite frozen storage of extracts for up to 9 months, spectra acquired on the NIH instrument (Center 1) were equivalent to or better than results obtained originally using fresh extracts on seven of nine non-NIH instruments (Table 3). This indicated that protein extraction quality was not the limiting factor, suggesting that an important parameter contributing to result variability was differences between instruments.

**TABLE 3:** Evaluation of instructional video and extract quality (*n* = 80 isolates).

**Center**	**NIH Mold Database at Score ≥ 2.0 (% accuracy)**			
	**Original center (video procedure)**	**Frozen extracts analyzed on NIH instrument (time stored at −20°C prior to analysis)**	***p*-Value Fresh extraction following instructional video Original center (Center 1 vs. Centers 2–8)**	***p*-Value Frozen extracts run on original center instrument vs. NIH instrument (after 1–9 months frozen)**
1	71	61 (9 months)	NA	0.4
2A^∗^	19	19 (9 months)	<0.0001	1.000
2B^∗^	22	19 (9 months)	<0.0001	0.7
2C^∗^	29∼	19 (9 months)	<0.0001	0.2
3	51	75 (8 months)	0.01	0.003
4	53	67 (6 months)	0.03	0.1
5	51	75 (1 month)	0.02	0.004
6	36	60 (6 months)	<0.0001	0.003
7	51	59 (3 months)	0.02	0.4
8	34	46 (6 months)	<0.0001	0.2

*^∗^Center 2 ran extracts on three instruments (A-C) after watching the video. ∼Original performance success was 7% and the instrument failed a few days later requiring preventative maintenance and part replacement. Data shown here is from the same extracts tested on the instrument after service and part replacement.*

### Instrument Optimization Alone Was Not Sufficient to Obtain Optimal Identification

Because spectra acquired on the NIH instrument continued to demonstrate better performance regardless of the origin of the protein extract, remote instrument service sessions were conducted with two participating centers in a pilot analysis to evaluate the effect of readjusting instrument settings such as the detector gain, ion source voltage, lens voltage, pulsed ion extraction time, and laser attenuator offset beyond adjustments made during routine preventative maintenance visits. Potential differences in extraction technique were controlled through the distribution of fresh frozen suspensions that had been extracted at the NIH. No significant difference was observed after instrument optimization in that specific circumstance (Supplementary Table 1). Furthermore, during follow up investigation, spectra acquired directly from fresh frozen extracts using the standard MBT_AutoX method on an instrument at Bruker US headquarters illustrated reproducibility of NIH results (100 vs. 94%, *p*-value 1.0; Supplementary Table 1), demonstrating the possibility of equivalent performance on a non-NIH instrument using the NIH database. This suggested that additional parameters, other than those that had been adjusted in this pilot analysis, may be critical for optimal mold identification. Notably, the instrument at Bruker US headquarters was used less frequently than instruments in clinical labs that run many samples per day on a continuous basis. This may have contributed to a state of better overall maintenance and thus better spectral quality and reproducibility. At several institutions, differences in performance were noted over time on a single instrument (Supplementary Figure 3).

### Identification of Key Spectral Acquisition and Processing Parameters for Optimized Mold Identification

A side-by-side comparison of spectral acquisition and processing parameters identified three key settings that repeatedly played a role in performance success in many cases (Supplementary Table 2). Modification of settings from the default Bruker MBT_AutoX method (used routinely for bacterial and yeast identification) to the settings of the NIH acquisition method or to those of an alternative method (Alternate-B method) based on this analysis, demonstrated improvement in some cases in a small-scale (six extracts) pilot analysis at five non-NIH study sites (Supplementary Figure 4). For some centers, the change in acquisition method resulted in significant improvement. For Center 5, only a change to the NIH acquisition method resulted in improved performance; equivalent performance was observed with the Alternate-B method.

**FIGURE 1:**
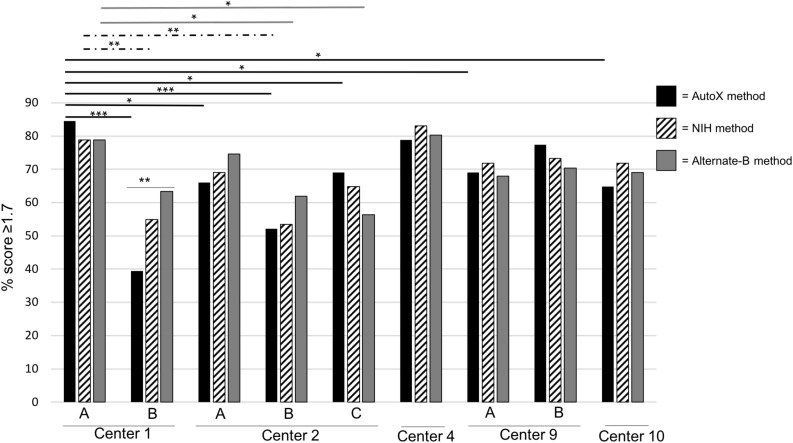
Comparative Analysis of Three Different Spectral Acquisition Methods Using a Single Prepared Plate (2018). Fresh extracts for 71 isolates were prepared at Center 1 and distributed to each institution. For each instrument, a freshly spotted plate was made and spectra were acquired on the same day in this order of spectral acquisition methods: (1) MBT_AutoX method (black bars and lines); (2) NIH method (hashed bars and lines); and (3) Alternate-B method (gray bars and lines). Multiple instruments in a single center are denoted by letters. ^∗^*p* < 0.05, ^∗∗^*p* < 0.005, ^∗∗∗^*p* < 0.0005.

To verify that use of the NIH method and the Alternate-B method could improve mold identification, a final challenge analysis was undertaken. Here, the full set of 80 isolates were subbed fresh and re-extracted at Center 1. 71 organisms remained viable from which frozen extracts were sent to three of the original testing centers, and two additional study sites (total of nine microflex LT instruments). Detailed instructions and screenshots for programming the NIH method and Alternate-B method were distributed to each center (Supplementary Figures 1, 2) and the sample plate, at each center, was run consecutively using each of the three acquisition methods (MBT_AutoX, NIH method, and Alternate-B method). A freshly spotted plate was used for each instrument. Although statistical significance between acquisition methods was achieved only on instrument 1B (Figure 1), wide performance variability remained across instruments for the MBT_AutoX acquisition method (39–85%, *p*-value 0.0001). Intra-laboratory variability was also observed in Centers 1, 2, and 9 where multiple instruments were tested despite controlling for operator, isolates, technique, extraction, and methods within each respective institution. This demonstrates that multiple controllable variables contribute to the successful and reproducible performance of MALDI-TOF MS for mold identification but that instrument age and condition may also contribute to variation observed between centers and between instruments within a single center. Data suggests that the amount of improvement observed with the use of modified acquisition methods might be greater if instrument performance is not optimized (for example, contrast Centers 3 and 7 in Supplementary Figure 4; and Centers 1B and 4 in Figure 1). We propose that evaluation on a larger and diverse fungal dataset on many instruments would confirm these findings.

## Discussion

**FIGURE 2:**
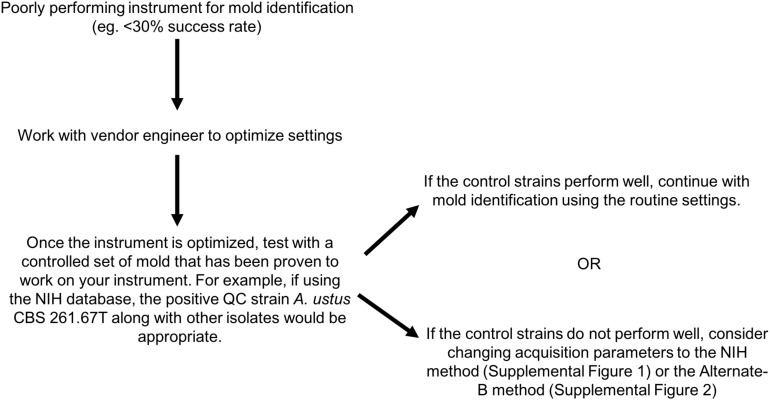
Optimization guide for laboratories experiencing low-level performance for mold identification by MALDI-TOF MS.

MALDI-TOF MS is an excellent tool for the rapid and accurate identification of molds (Alanio et al., 2011; Cassagne et al., 2011; Theel et al., 2011; Alshawa et al., 2012; De Carolis et al., 2012; Del Chierico et al., 2012; Schrodl et al., 2012; de Respinis et al., 2013; Lau et al., 2013; L’Ollivier et al., 2013; Nenoff et al., 2013; Normand et al., 2013, 2017; Barker et al., 2014; Becker et al., 2014, 2019; Gautier et al., 2014; Ranque et al., 2014; Sitterle et al., 2014; Dolatabadi et al., 2015; Triest et al., 2015; Sleiman et al., 2016; Stein et al., 2018; Dupont et al., 2019), yet its wider application in the clinical setting has been extremely limited, in part due to limited database availability and lack of standardized processes. Here, we evaluated the versatility of the NIH Mold Database (Lau et al., 2013) across 10 centers each with varying levels of mass spectrometry experience with molds, using a set of 80 challenge isolates that encompassed common clinical molds and less common organisms (Table 1). Figure 2 provides a summary of optimization steps that laboratories may apply if mold identification using the routine setting on their instrument is suboptimal.

The wide performance variation between centers demonstrated in this study (Figure 1 and Tables 2, 3) was surprising given that high inter- and intra-laboratory reproducibility has been achieved for bacterial and yeast studies (Westblade et al., 2015; Wilson et al., 2017). We showed, however, that application of different spectral acquisition parameters (NIH and Alternate-B methods), along with optimized instrument tuning and maintenance, improved performance in several cases (Figure 1 and Supplementary Figure 4), suggesting that acquisition parameters are one of the key limiting variables in achieving successful performance. Comparison of acquisition methods (Supplementary Table 2) demonstrated that the minimum intensity threshold varied considerably between the MBT_AutoX, NIH, and Alternate-B methods. This parameter relates to acceptability criteria for peptide and protein peaks from molds, which are more difficult to lyse than bacteria and yeast. Interestingly, Normand et al. (2017) also identified wide performance variation between five sites that evaluated an online database for spectra acquired by the Bruker MBT system. In that study, differences were attributed to variations in sample preparation, matrix quality, and functions of the different mass spectrometers (same microflex LT model but different age and usage). Similar intra-instrument variability findings and inherent reproducibility problems were also found in a Canadian multicenter study (Stein et al., 2018). These previous publications differ to ours because they challenged different databases and studied incoming real-time samples as opposed to a standardized comparative set as used in this study. Furthermore, extraction technique and operator experience were also controlled for in our study for defined experiments. Therefore, the reproducibility of inter- and intra-instrument variability observed now across several multicenter studies that target different aspects of the process clearly suggests that MALDI-TOF MS (at least on the Bruker platform) requires optimization for mold identification, and that spotting in replicates (up to quadruplicates as proposed by Gautier et al., 2014) is not ideal. Here, we show for the first time that optimizing acquisition parameters may potentially reduce inter-operator and inter-instrument variation. In fact, the most recently released Bruker RUO software (v3.0, August 2019) has provided adjusted acquisition parameters and adapted thresholds to aid in mold identification. Given its recent availability, it is yet to be seen whether these manufacturer updates will provide improved performance for mold identification within the clinical setting. In-house developed databases using the Bruker MBT system were applied in this current study and in Normand et al. (2017). In contrast, Rychert et al. (2018) reported 94% reproducibility across three centers that evaluated the VITEK MS 3.0 (bioMerieux) platform. Importantly, the challenge sets studied in each of the multicenter evaluations varied considerably with Rychert et al. (2018) employing 50 very common clinical molds in contrast to the wider isolate diversity used in this study (Table 1) and in Normand et al. (2017). Organism challenge sets are an important consideration for reproducibility assessment and performance evaluation because MSPs for common clinical isolates are more likely to be sufficiently represented compared with rarer organisms.

While we have identified that use of modified acquisition parameters can improve performance, it is clear that other variables, potentially source cleanliness, and age of the laser and instrument components, can also affect performance (Supplementary Figure 3). These variables are instrument dependent and may require individual system optimization in addition to routine instrument calibration and maintenance. Development of a performance/calibration standard that is specific for molds and is tested during routine maintenance may be useful for instrument programming to ensure reproducibility. We propose that the *A. ustus* CBS 261.67T control strain be used as a marker for instrument performance, with optimal instrument performance leading to consistent log scores > 2.0 for this control strain when used with the NIH Mold Database (Lau et al., 2013), even though we have demonstrated that lowering the threshold to ≥ 1.7 for unknown patient isolates improves sensitivity without affecting accuracy. Failure of an instrument to meet these criteria would be an indication for maintenance and performance optimization. Many laboratories do not have microbiologists familiar with changing spectral acquisition parameters, and an easy to use validated method provided by the manufacturer would be ideal.

The slow adoption of MALDI-TOF MS for mold identification has been associated with the lack of supporting data and practical methods. Few investigators have evaluated the Bruker Filamentous Fungi database using the Bruker recommended protocol of liquid fungal cultures (Schulthess et al., 2014) which is not convenient for integration into routine workflow. Evaluation of the VITEK MS 3.0 (bioMerieux) has demonstrated 66.8 to 91% identification accuracy in the clinical setting, with some concerns for misidentification (McMullen et al., 2016; Rychert et al., 2018). Based on these limited data, it is not surprising that most efforts have focused on laboratory-developed databases which have often outperformed those of the manufacturer (Alanio et al., 2011; Cassagne et al., 2011; Theel et al., 2011; Alshawa et al., 2012; De Carolis et al., 2012; Del Chierico et al., 2012; Schrodl et al., 2012; de Respinis et al., 2013; Lau et al., 2013; L’Ollivier et al., 2013; Nenoff et al., 2013; Normand et al., 2013, 2017; Barker et al., 2014; Becker et al., 2014; Gautier et al., 2014; Ranque et al., 2014; Sitterle et al., 2014; Dolatabadi et al., 2015; Triest et al., 2015; Sleiman et al., 2016). A major limitation, however, is that all but a few studies such as this study, Normand et al. (2017), Rychert et al. (2018), and Stein et al. (2018) have been single-institution (and single-instrument) analyses that have not addressed the significant reproducibility issues highlighted in this study. With increased implementation of MALDI-TOF MS for routine organism identification in laboratories, availability of an FDA cleared database (bioMerieux) and open access databases (Lau et al., 2013; Normand et al., 2017; Center for Disease Control and Prevention [CDCP], 2019), and with the provision of alternative spectral acquisition methods for Bruker Biotyper instruments described in this study (Supplementary Figures 1, 2) and those spectral acquisition modifications recently released by the manufacturer in updated software (v3.0), it is likely that rapid and accurate mold identification using MALDI-TOF MS can become a norm in clinical laboratories.

## Conclusion

We have shown that the NIH Mold MALDI-TOF MS Database can be successfully transferred and implemented as a routine method for mold identification across multiple institutions if alternate spectral acquisition methods are applied along with optimization of instrument performance. A clinical laboratory initiating use of MALDI-TOF MS for mold identification would become frustrated with performance of < 30% success (e.g., Center 2A in Supplementary Figure 3), but should be reassured that > 80% success (as exemplified multiple times in this study) is possible with optimization, and they should be encouraged to work persistently with the vendor engineer until their instrument performs equivalently to those illustrated here. Continued vigilance is necessary to assure that instrument performance is maintained at the optimized level because performance can be affected by instrument use and maintenance over time. Importantly, in our experience, as instrument optimization changes over time, the result is a low score preventing mold identification rather than inaccurate identifications. Refer to Figure 2 for stepwise guide for optimizing instrument performance. More rapid and accurate identification leads to better guidance for clinicians in the selection of antifungal therapy and the collection of epidemiological data. Analysis against a larger and diverse challenge set on instruments at additional institutions is warranted. Because instrument variability is inevitable and some parameters are instrument dependent, development of an instrument performance standard specific for mold spectral acquisition is suggested to improve reproducibility across instruments.

## Data Availability

All datasets generated for this study are included in the manuscript and/or the Supplementary Files.

## Author Contributions

AL and KF designed the study. AL, RW, HM, ES, KK, KR, AR, JG, KM, MW, ALB, UD, and CH performed the experiments and provided the data. AL, J-MB-L, BB, C-AB, SB-W, CD, KH, NL, PP, AS, NW, SZ, AZ, and KF analyzed the data and participated in group discussions. AB, MK, TM, and GS provided technical expertise on the instrument and assisted with design of the Alternate-B acquisition method. AL wrote the manuscript. All authors reviewed and edited the manuscript.

## Conflict of Interest Statement

AL, AZ, and KF were previously part of a collaborative agreement with Bruker Daltonics, Inc. for the development of microbial databases. KR, BB, and NL have received research funding from both Bruker Daltonics, Inc. and bioMerieux to conduct clinical trials. PP has received research funding from Bruker Daltonics, Inc. to conduct clinical trials. C-AB has received research funding from bioMerieux and speaker fees from Bruker Daltonics, Inc. AB, MK, TM, and GS are employees of Bruker Daltonics, Inc., the manufacturer of the MALDI-TOF MS system used in this study; Bruker Daltonics, Inc. had no influence on the design of the study, data collection, or the interpretation of the study results. The remaining authors declare that the research was conducted in the absence of any commercial or financial relationships that could be construed as a potential conflict of interest.

## Footnotes

1 https://www.dropbox.com/s/gt4mfk5nj5y9qif/NIH Mold MALDI Extraction Video.mov

2 www.graphpad.com
