# Supplementary material for: Multicenter Study Demonstrates Standardization Requirements for Mold Identification by MALDI-TOF MS
Source: Front Microbiol. 2019 Sep 20;10:2098. doi: 10.3389/fmicb.2019.02098 (PMC6764242; doi:10.3389/fmicb.2019.02098)
Supplement: Supplementary file 1 [file Image_1.pdf]

NIH
Spectral Acquisition Method

Supplemental Figure 1

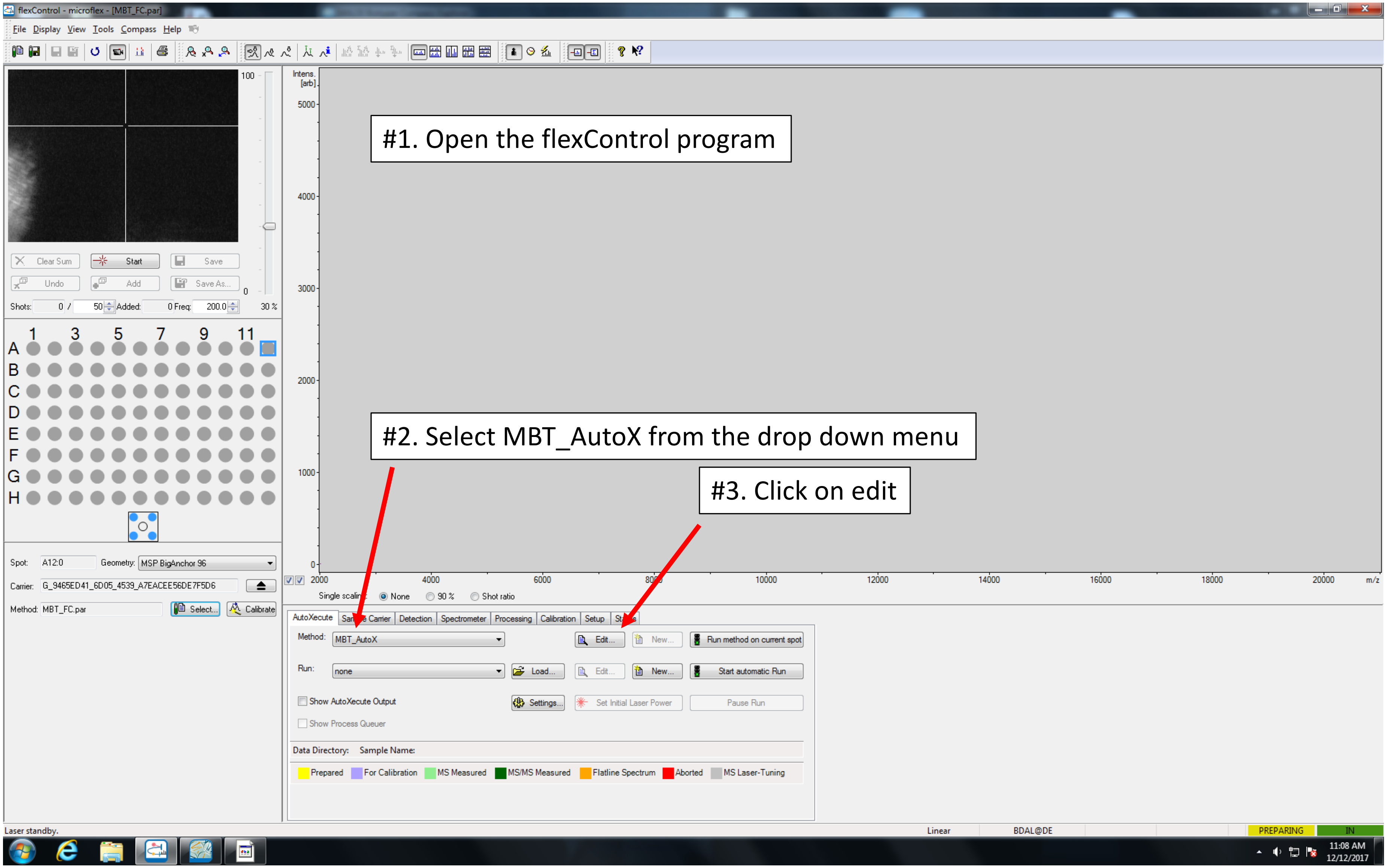
#1. Open the flexControl program
#2. Select MBT_AutoX from the drop down menu
#3. Click on edit
flexControl - microflex - [MBT_FC.par]
Method: MBT_AutoX
Edit...

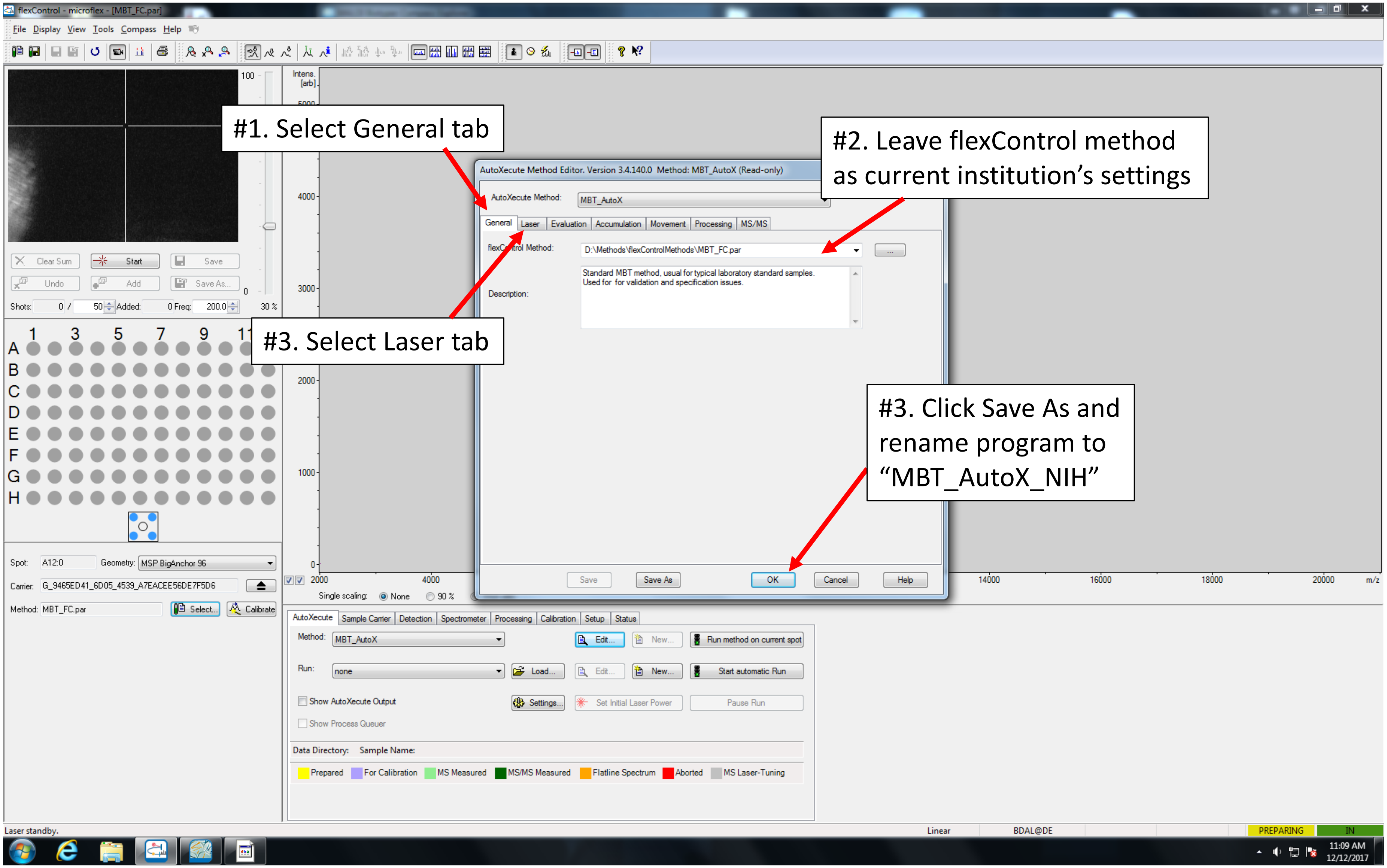
#1. Select General tab
#2. Leave flexControl method as current institution's settings
#3. Select Laser tab
#3. Click Save As and rename program to "MBT_AutoX_NIH"
AutoXecute Method Editor. Version 3.4.140.0 Method: MBT_AutoX (Read-only)
AutoXecute Method:
MBT_AutoX
General
Laser
Evaluation
Accumulation
Movement
Processing
MS/MS
D:\Methods\flexControlMethods\MBT_FC.par
Description:
Standard MBT method, usual for typical laboratory standard samples.
Used for for validation and specification issues.
Save
Save As
OK
Cancel
Help

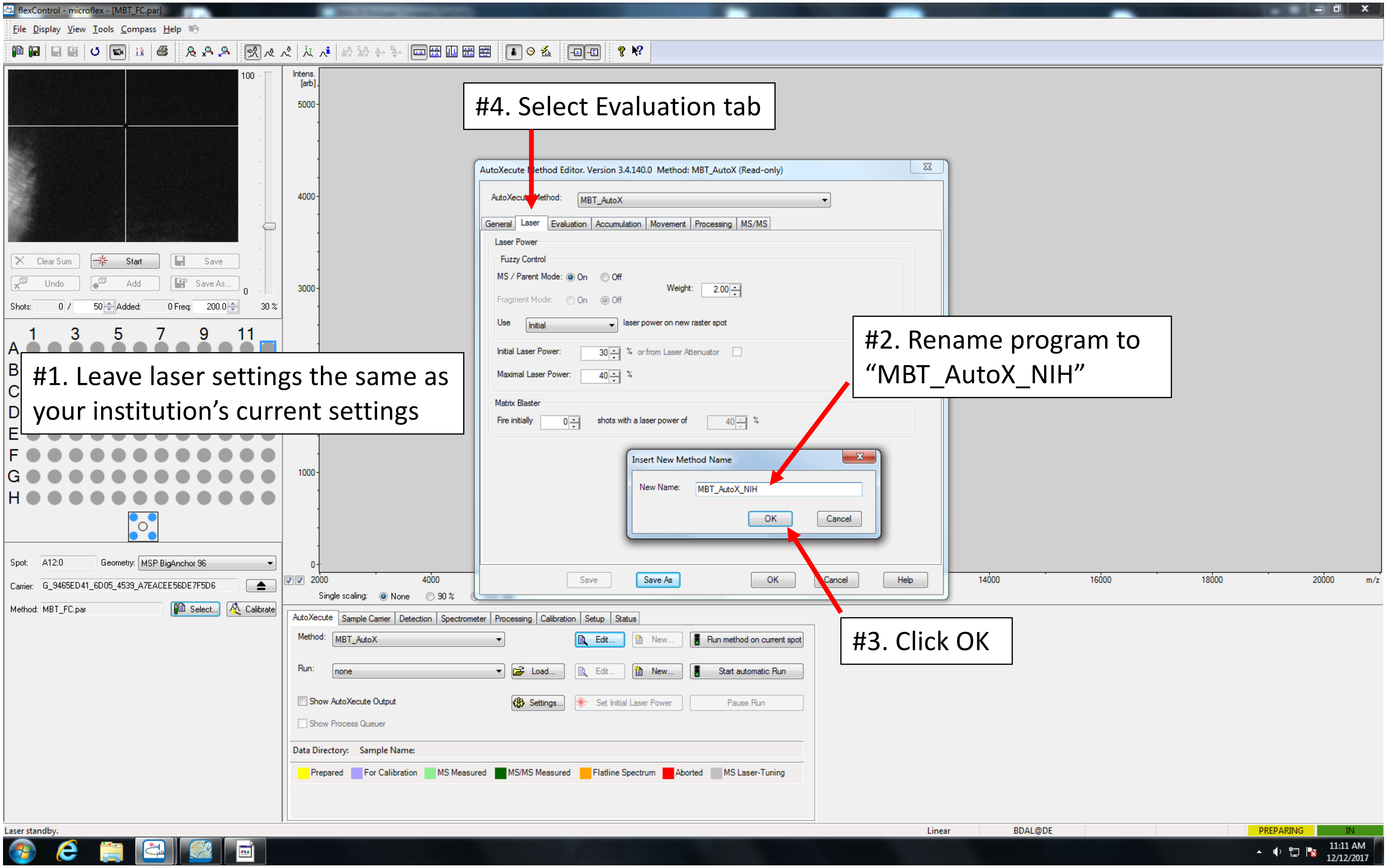
#4. Select Evaluation tab
#1. Leave laser settings the same as your institution's current settings
#2. Rename program to "MBT_AutoX_NIH"
#3. Click OK
Insert New Method Name
New Name:
MBT_AutoX_NIH
OK
Cancel

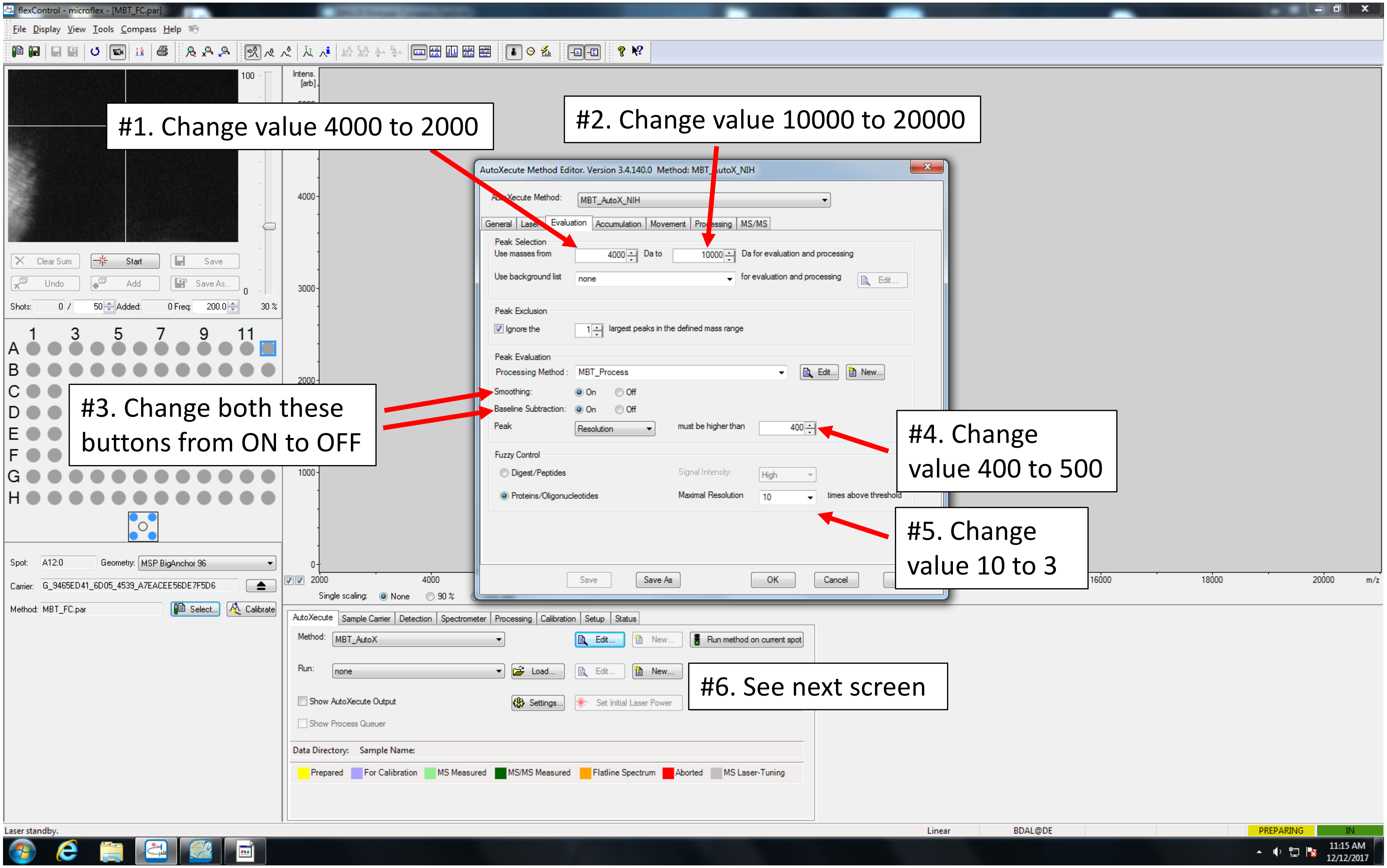
#1. Change value 4000 to 2000
#2. Change value 10000 to 20000
#3. Change both these buttons from ON to OFF
#4. Change value 400 to 500
#5. Change value 10 to 3
#6. See next screen
AutoXecute Method Editor. Version 3.4.140.0 Method: MBT_AutoX_NIH
AutoXecute Method:
MBT_AutoX_NIH
General
Evaluation
Accumulation
Movement
MS/MS
Peak Selection
Use masses from
4000
Da to
10000
Da for evaluation and processing
Use background list
none
for evaluation and processing
Peak Exclusion
Ignore the
1
largest peaks in the defined mass range
Peak Evaluation
Processing Method :
MBT_Process
Smoothing:
Baseline Subtraction:
On
Off
Peak
Resolution
must be higher than
400
Fuzzy Control
Digest/Peptides
Proteins/Oligonucleotides
Signal Intensity:
High
Maximal Resolution
10
times above threshold
Save
Save As
OK
Cancel

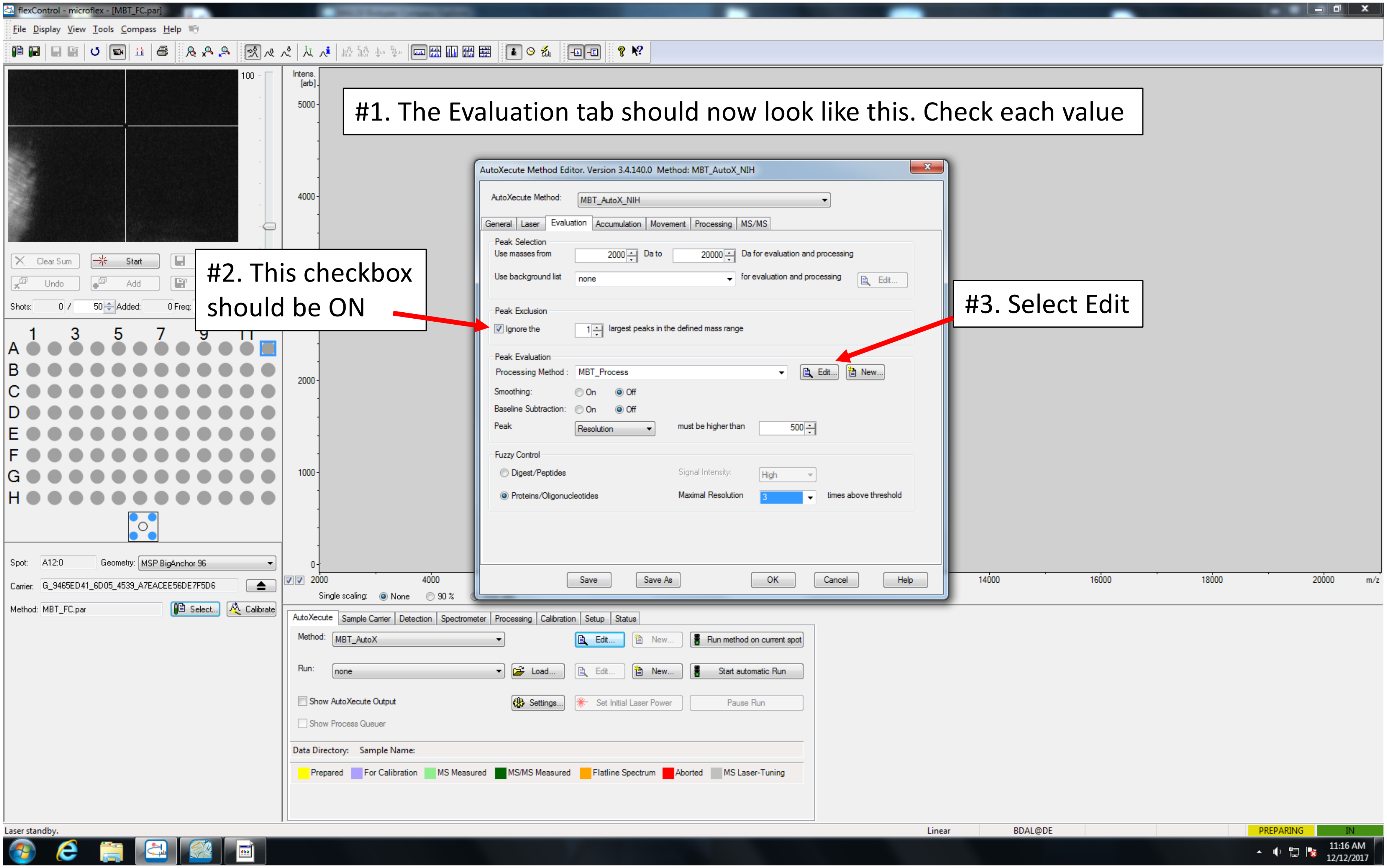
#1. The Evaluation tab should now look like this. Check each value
#2. This checkbox should be ON
#3. Select Edit
AutoXecute Method Editor. Version 3.4.140.0 Method: MBT_AutoX_NIH
AutoXecute Method: MBT_AutoX_NIH
General
Laser
Evaluation
Accumulation
Movement
Processing
MS/MS
Peak Selection
Use masses from 2000 Da to 20000 Da for evaluation and processing
Use background list none for evaluation and processing
Peak Exclusion
Ignore the 1 largest peaks in the defined mass range
Peak Evaluation
Processing Method : MBT_Process
Edit...
New...
Smoothing: On Off
Baseline Subtraction: On Off
Peak Resolution must be higher than 500
Fuzzy Control
Digest/Peptides
Proteins/Oligonucleotides
Signal Intensity: High
Maximal Resolution 3 times above threshold
Save
Save As
OK
Cancel
Help

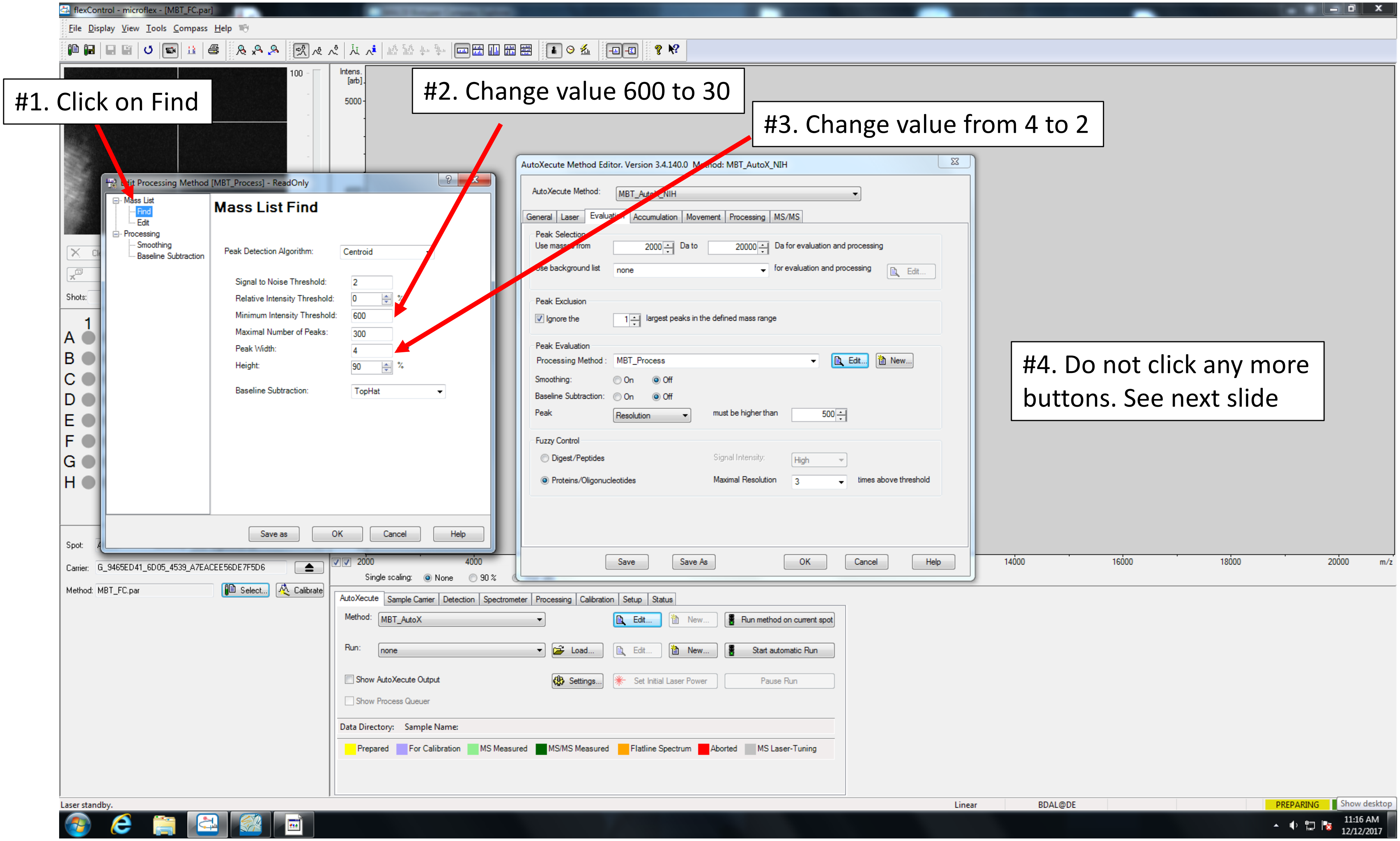
#1. Click on Find
#2. Change value 600 to 30
#3. Change value from 4 to 2
#4. Do not click any more buttons. See next slide
Edit Processing Method [MBT_Process] - ReadOnly
Mass List Find
Peak Detection Algorithm: Centroid
Signal to Noise Threshold: 2
Relative Intensity Threshold: 0
Minimum Intensity Threshold: 600
Maximal Number of Peaks: 300
Peak Width: 4
Height: 90
Baseline Subtraction: TopHat
AutoXecute Method Editor. Version 3.4.140.0 Method: MBT_AutoX_NIH

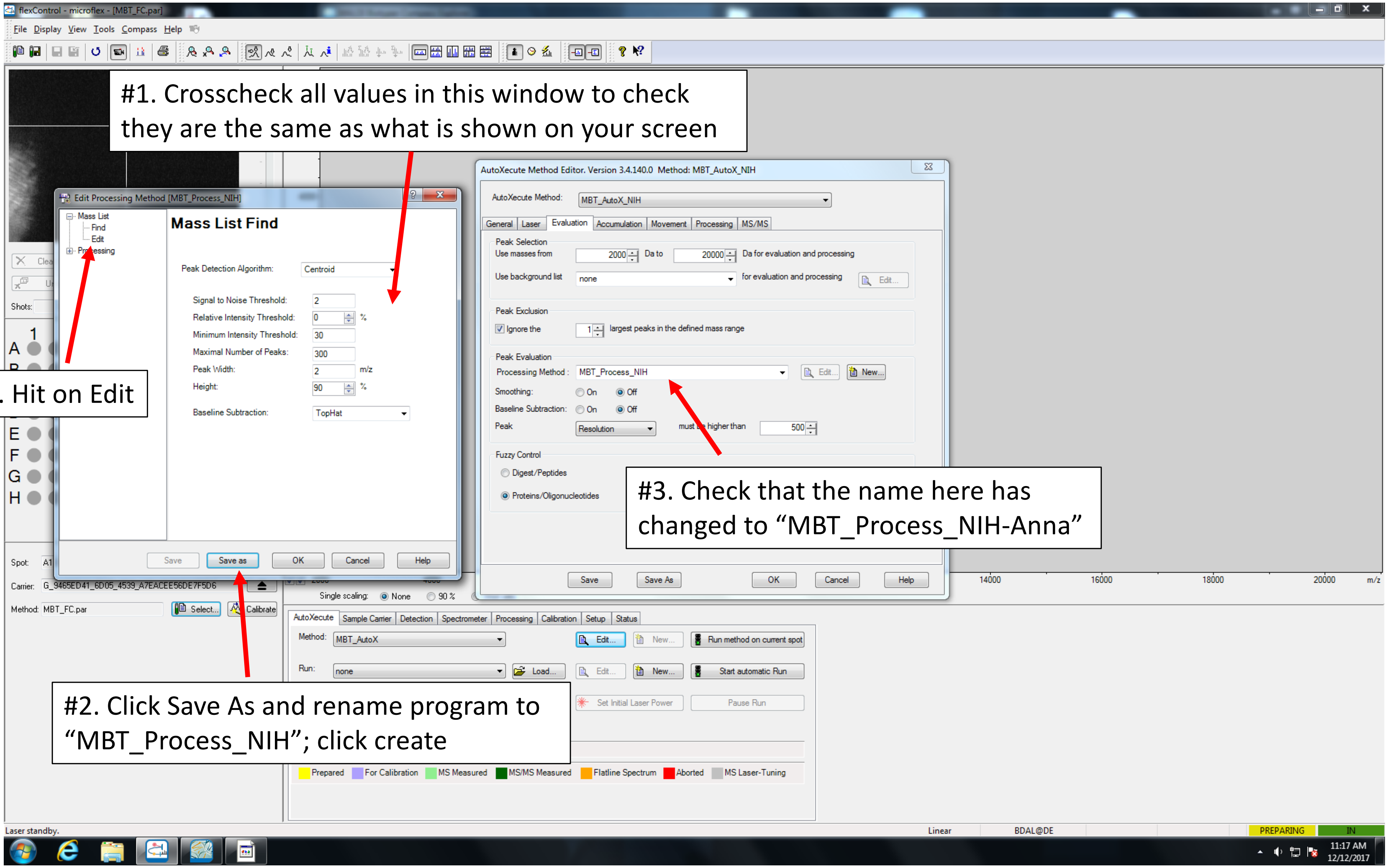

#1. Crosscheck all values in this window to check they are the same as what is shown on your screen
#2. Click Save As and rename program to "MBT_Process_NIH"; click create
#3. Check that the name here has changed to "MBT_Process_NIH-Anna"
#4. Hit on Edit
flexControl - microflex - [MBT_FC.par]
Edit Processing Method [MBT_Process_NIH]
Mass List Find
Peak Detection Algorithm: Centroid
Signal to Noise Threshold: 2
Relative Intensity Threshold: 0 %
Minimum Intensity Threshold: 30
Maximal Number of Peaks: 300
Peak Width: 2 m/z
Height: 90 %
Baseline Subtraction: TopHat
Save
Save as
OK
Cancel
Help
AutoXecute Method Editor. Version 3.4.140.0 Method: MBT_AutoX_NIH
AutoXecute Method: MBT_AutoX_NIH
General
Laser
Evaluation
Accumulation
Movement
Processing
MS/MS
Peak Selection
Use masses from 2000 Da to 20000 Da for evaluation and processing
Use background list none for evaluation and processing
Peak Exclusion
Ignore the 1 largest peaks in the defined mass range
Peak Evaluation
Processing Method: MBT_Process_NIH
Smoothing: On Off
Baseline Subtraction: On Off
Peak Resolution must be higher than 500
Fuzzy Control
Digest/Peptides
Proteins/Oligonucleotides
Save As
Method: MBT_AutoX
Run: none
Edit...
New...
Run method on current spot
Load...
Start automatic Run
Set Initial Laser Power
Pause Run
Prepared
For Calibration
MS Measured
MS/MS Measured
Flatline Spectrum
Aborted
MS Laser-Tuning
Laser standby.

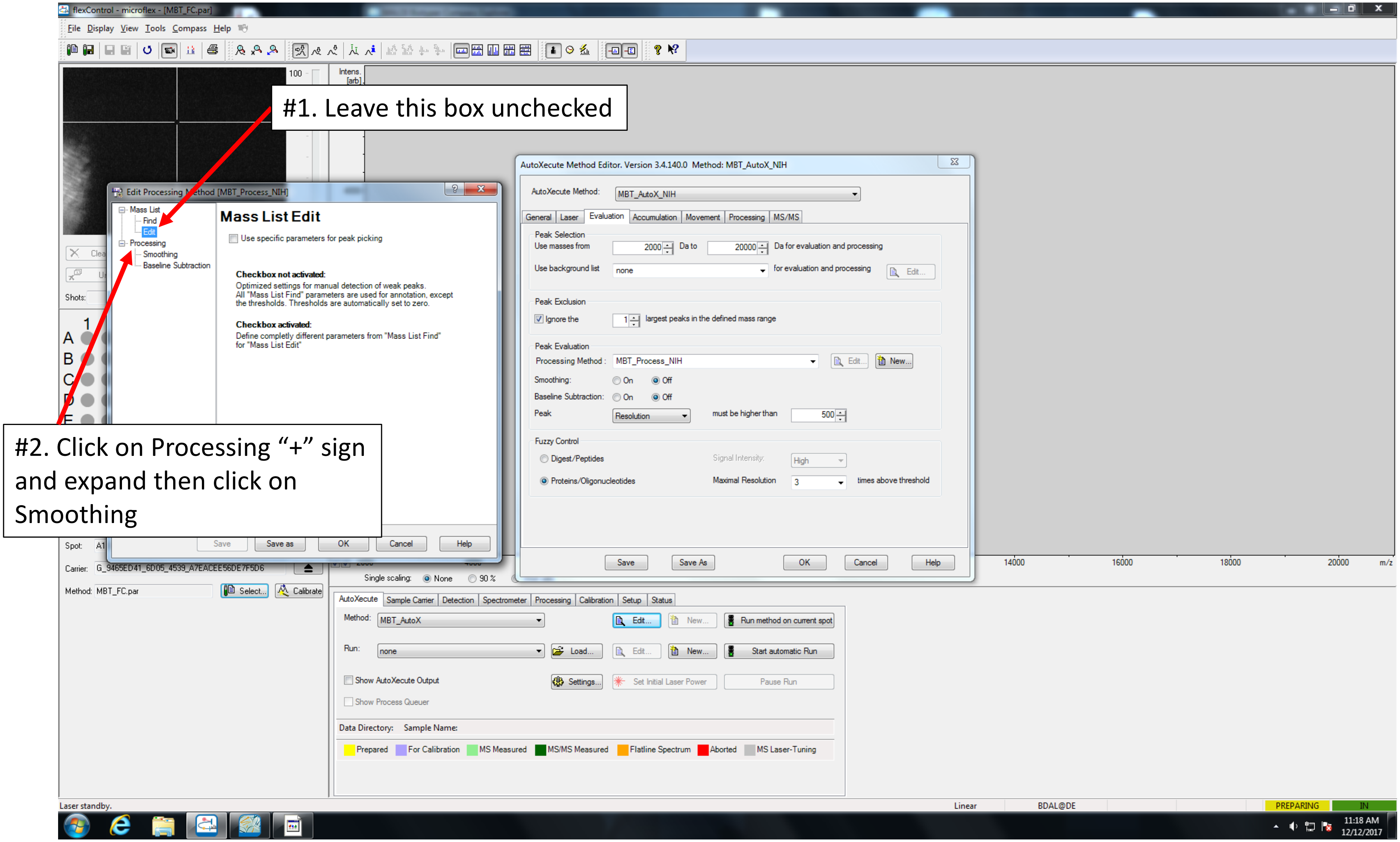
#1. Leave this box unchecked
#2. Click on Processing "+" sign and expand then click on Smoothing
flexControl - microflex - [MBT_FC.par]
File Display View Tools Compass Help
Edit Processing Method [MBT_Process_NIH]
Mass List
Find
Edit
Processing
Smoothing
Baseline Subtraction
Mass List Edit
Use specific parameters for peak picking
Checkbox not activated:
Optimized settings for manual detection of weak peaks.
All "Mass List Find" parameters are used for annotation, except the thresholds. Thresholds are automatically set to zero.
Checkbox activated:
Define completly different parameters from "Mass List Find" for "Mass List Edit"
Save
Save as
OK
Cancel
Help
AutoXecute Method Editor. Version 3.4.140.0 Method: MBT_AutoX_NIH
AutoXecute Method:
MBT_AutoX_NIH
General
Laser
Evaluation
Accumulation
Movement
Processing
MS/MS
Peak Selection
Use masses from
2000
Da to
20000
Da for evaluation and processing
Use background list
none
for evaluation and processing
Peak Exclusion
Ignore the
1
largest peaks in the defined mass range
Peak Evaluation
Processing Method :
MBT_Process_NIH
Smoothing:
On
Off
Baseline Subtraction:
On
Off
Peak
Resolution
must be higher than
500
Fuzzy Control
Digest/Peptides
Signal Intensity:
High
Proteins/Oligonucleotides
Maximal Resolution
3
times above threshold
Save
Save As
OK
Cancel
Help
AutoXecute
Sample Carrier
Detection
Spectrometer
Processing
Calibration
Setup
Status
Method:
MBT_AutoX
Run:
none
Run method on current spot
Start automatic Run
Show AutoXecute Output
Show Process Queuer
Settings...
Set Initial Laser Power
Pause Run
Data Directory:
Sample Name:
Prepared
For Calibration
MS Measured
MS/MS Measured
Flatline Spectrum
Aborted
MS Laser-Tuning
Carrier:
G_9465ED41_6D05_4539_A7EACEE56DE7F5D6
Method:
MBT_FC.par
Select...
Calibrate
Single scaling:
None
90 %
14000
16000
18000
20000
m/z
Laser standby.
Linear
BDAL@DE
PREPARING
11:18 AM
12/12/2017

#1. No edits on this screen. Confirm that values match what is shown here

#2. Click on Baseline subtraction

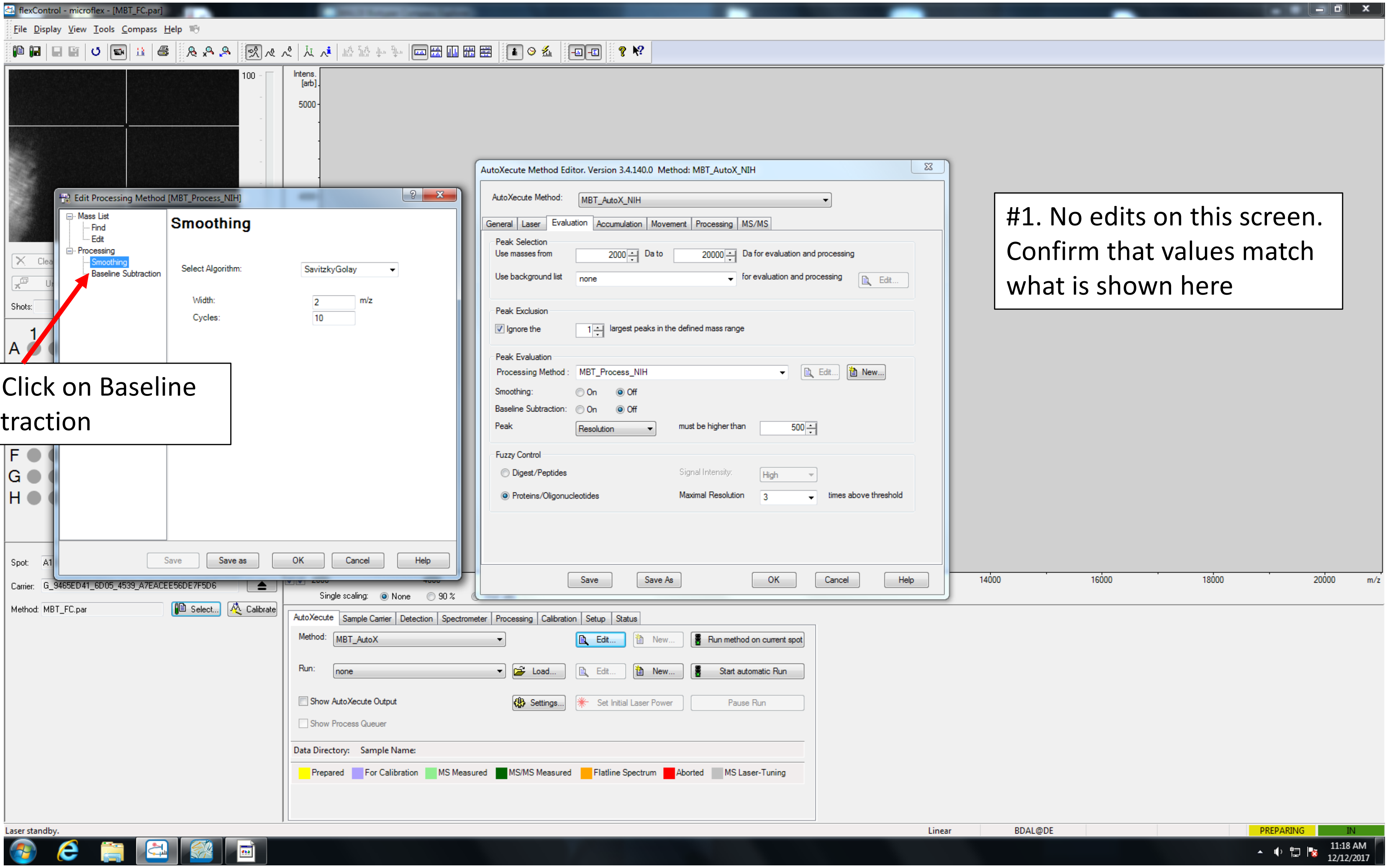

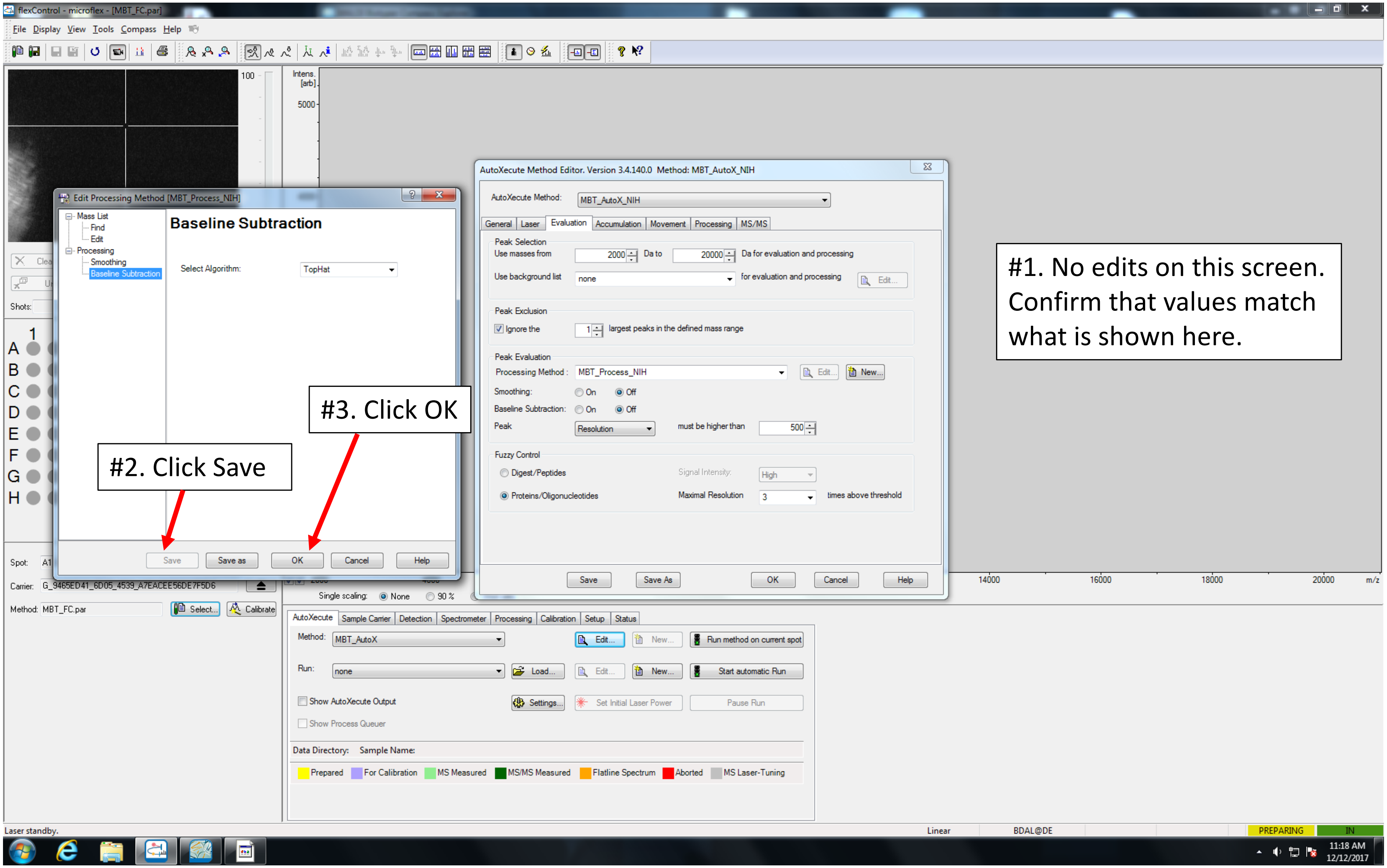
#1. No edits on this screen. Confirm that values match what is shown here.
#2. Click Save
#3. Click OK
Edit Processing Method [MBT_Process_NIH]
Baseline Subtraction
Select Algorithm:
TopHat
AutoXecute Method Editor. Version 3.4.140.0 Method: MBT_AutoX_NIH
AutoXecute Method:
MBT_AutoX_NIH
Peak Selection
Use masses from
2000
Da to
20000
Da for evaluation and processing
Use background list
none
for evaluation and processing
Peak Exclusion
Ignore the
1
largest peaks in the defined mass range
Peak Evaluation
Processing Method :
MBT_Process_NIH
Smoothing:
Baseline Subtraction:
Peak
Resolution
must be higher than
500
Fuzzy Control
Digest/Peptides
Proteins/Oligonucleotides
Signal Intensity:
High
Maximal Resolution
3
times above threshold

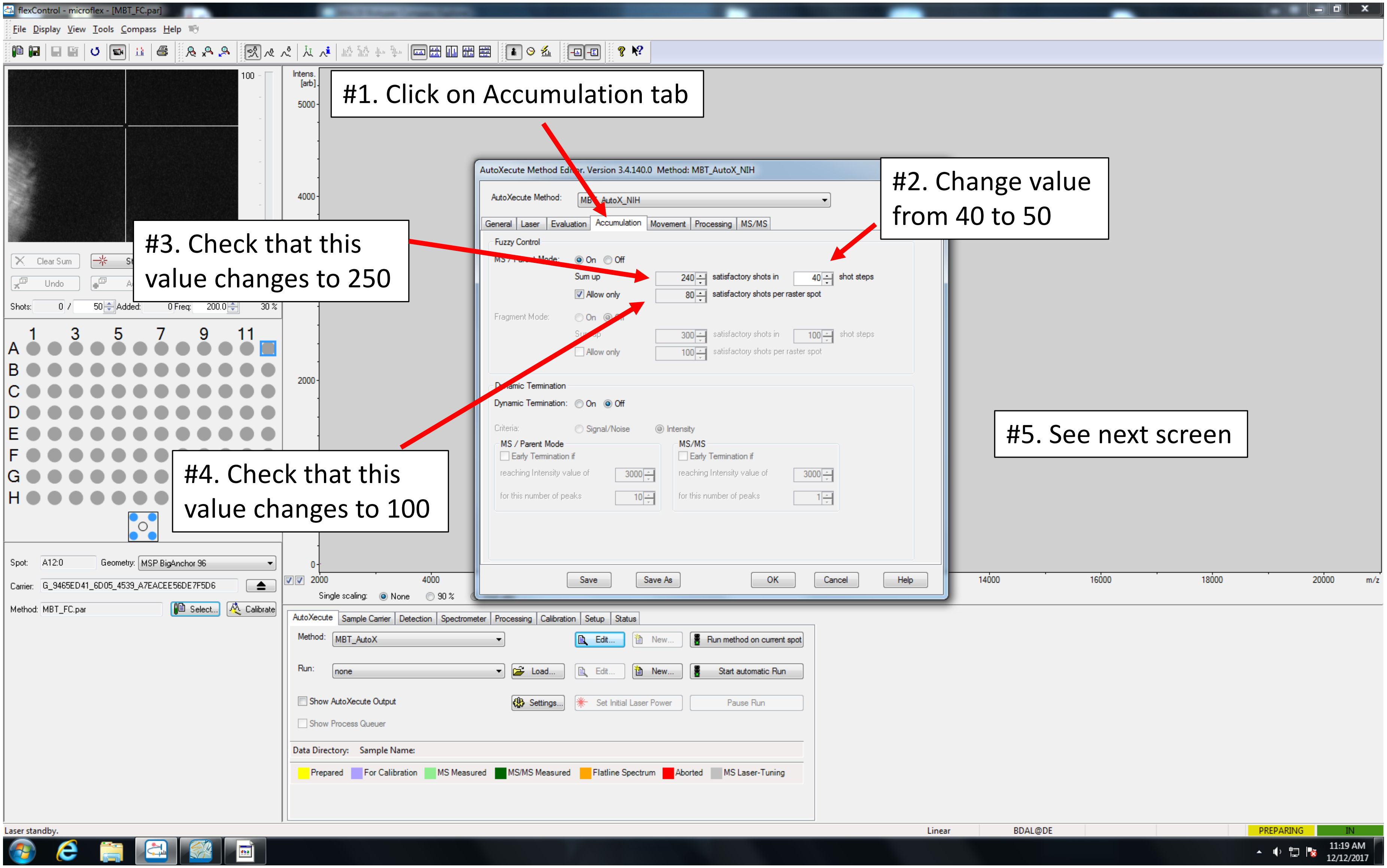
#1. Click on Accumulation tab
#2. Change value from 40 to 50
#3. Check that this value changes to 250
#4. Check that this value changes to 100
#5. See next screen
flexControl - microflex - [MBT_FC.par]
File Display View Tools Compass Help
AutoXecute Method Editor. Version 3.4.140.0 Method: MBT_AutoX_NIH
AutoXecute Method:
MBT_AutoX_NIH
General
Laser
Evaluation
Accumulation
Movement
Processing
MS/MS
Fuzzy Control
Sum up
240
satisfactory shots in
40
shot steps
Allow only
80
satisfactory shots per raster spot
Fragment Mode:
300
100
Dynamic Termination
Dynamic Termination: On Off
Criteria:
Signal/Noise
Intensity
MS / Parent Mode
Early Termination if
reaching Intensity value of
3000
for this number of peaks
10
MS/MS
Save
Save As
OK
Cancel
Help
Spot: A12:0
Geometry: MSP BigAnchor 96
Carrier: G_9465ED41_6D05_4539_A7EACEE56DE7F5D6
Method: MBT_FC.par
Select...
Calibrate
AutoXecute
Sample Carrier
Detection
Spectrometer
Calibration
Setup
Status
Method:
MBT_AutoX
Edit...
New...
Run method on current spot
Run:
none
Load...
Start automatic Run
Show AutoXecute Output
Settings...
Set Initial Laser Power
Pause Run
Show Process Queuer
Data Directory:
Sample Name:
Prepared
For Calibration
MS Measured
MS/MS Measured
Flatline Spectrum
Aborted
MS Laser-Tuning
Laser standby.
Linear
BDAL@DE
PREPARING
IN
11:19 AM
12/12/2017

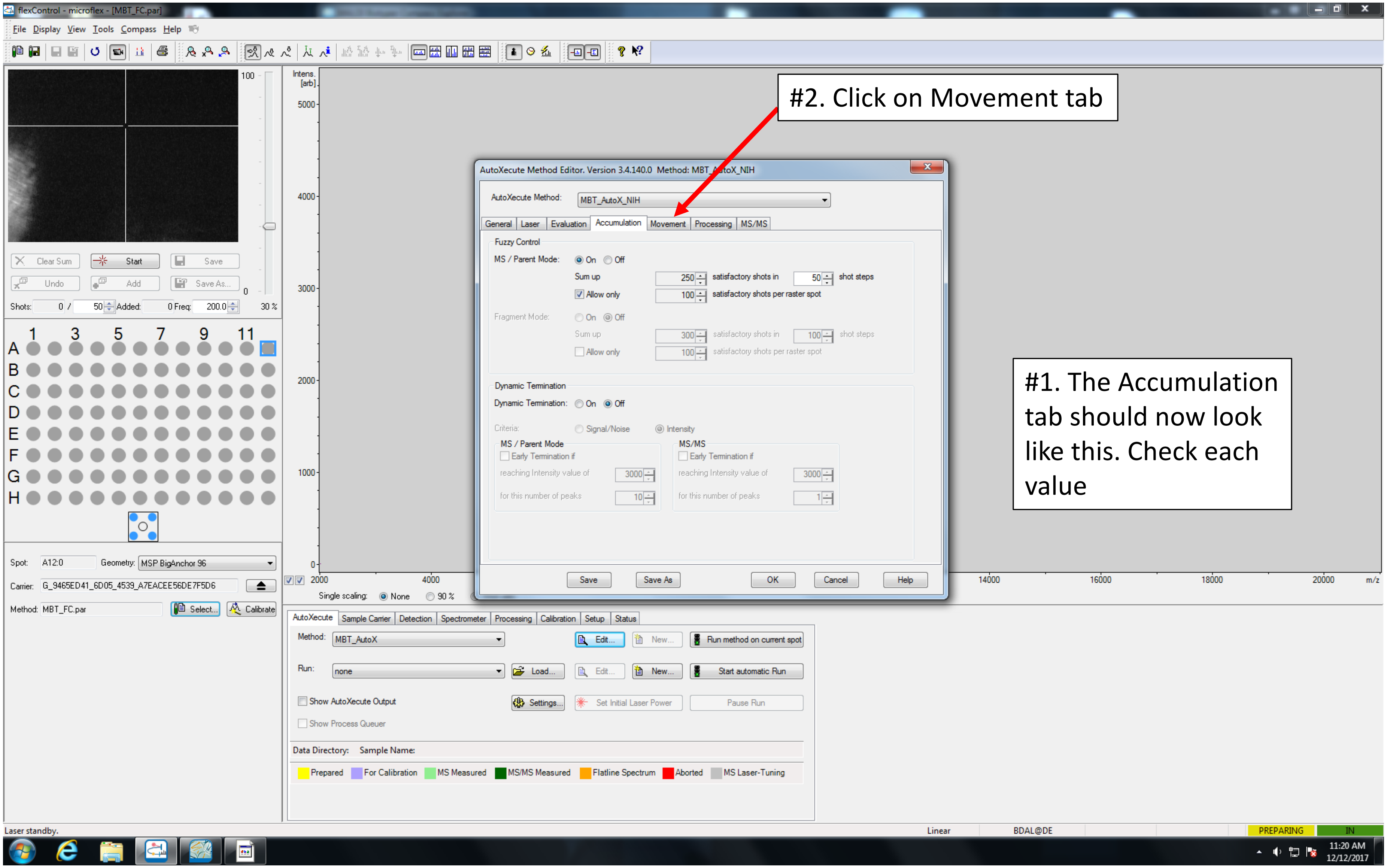
#2. Click on Movement tab
#1. The Accumulation tab should now look like this. Check each value
flexControl - microflex - [MBT_FC.par]
File Display View Tools Compass Help
AutoXecute Method Editor. Version 3.4.140.0 Method: MBT_AutoX_NIH
AutoXecute Method: MBT_AutoX_NIH
General Laser Evaluation Accumulation Movement Processing MS/MS
Fuzzy Control
MS / Parent Mode: On Off
Sum up 250 satisfactory shots in 50 shot steps
Allow only 100 satisfactory shots per raster spot
Fragment Mode: On Off
Sum up 300 satisfactory shots in 100 shot steps
Allow only 100 satisfactory shots per raster spot
Dynamic Termination
Dynamic Termination: On Off
Criteria: Signal/Noise Intensity
MS / Parent Mode
Early Termination if
reaching Intensity value of 3000
for this number of peaks 10
MS/MS
Early Termination if
reaching Intensity value of 3000
for this number of peaks 1
Save Save As OK Cancel Help
AutoXecute Sample Carrier Detection Spectrometer Processing Calibration Setup Status
Method: MBT_AutoX
Run: none
Edit... New... Run method on current spot
Load... Edit... New... Start automatic Run
Show AutoXecute Output
Show Process Queuer
Settings... Set Initial Laser Power Pause Run
Data Directory: Sample Name:
Prepared For Calibration MS Measured MS/MS Measured Flatline Spectrum Aborted MS Laser-Tuning
Spot: A12:0 Geometry: MSP BigAnchor 96
Carrier: G_9465ED41_6D05_4539_A7EACEE56DE7F5D6
Method: MBT_FC.par Select... Calibrate
Clear Sum Start Save
Undo Add Save As...
Laser standby.
Linear BDAL@DE PREPARING IN
11:20 AM 12/12/2017

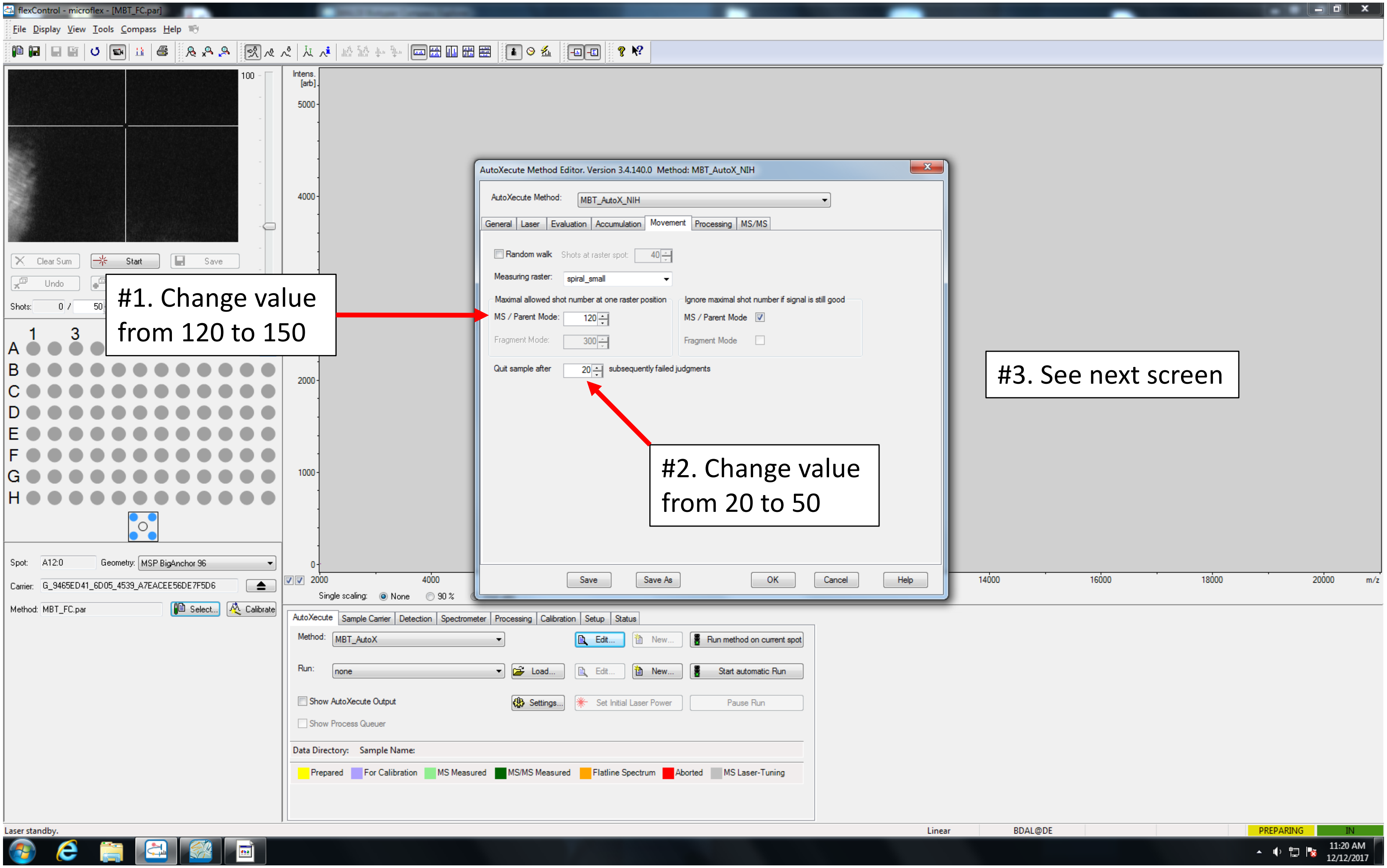

#1. Change value from 120 to 150
#2. Change value from 20 to 50
#3. See next screen
AutoXecute Method Editor. Version 3.4.140.0 Method: MBT_AutoX_NIH
AutoXecute Method: MBT_AutoX_NIH
General Laser Evaluation Accumulation Movement Processing MS/MS
Random walk
Shots at raster spot: 40
Measuring raster: spiral_small
Maximal allowed shot number at one raster position
MS / Parent Mode: 120
Fragment Mode: 300
Ignore maximal shot number if signal is still good
MS / Parent Mode
Fragment Mode
Quit sample after 20 subsequently failed judgments
Save
Save As
OK
Cancel
Help

#2. Click on Processing tab

#1. The Movement tab should now look like this. Check each value

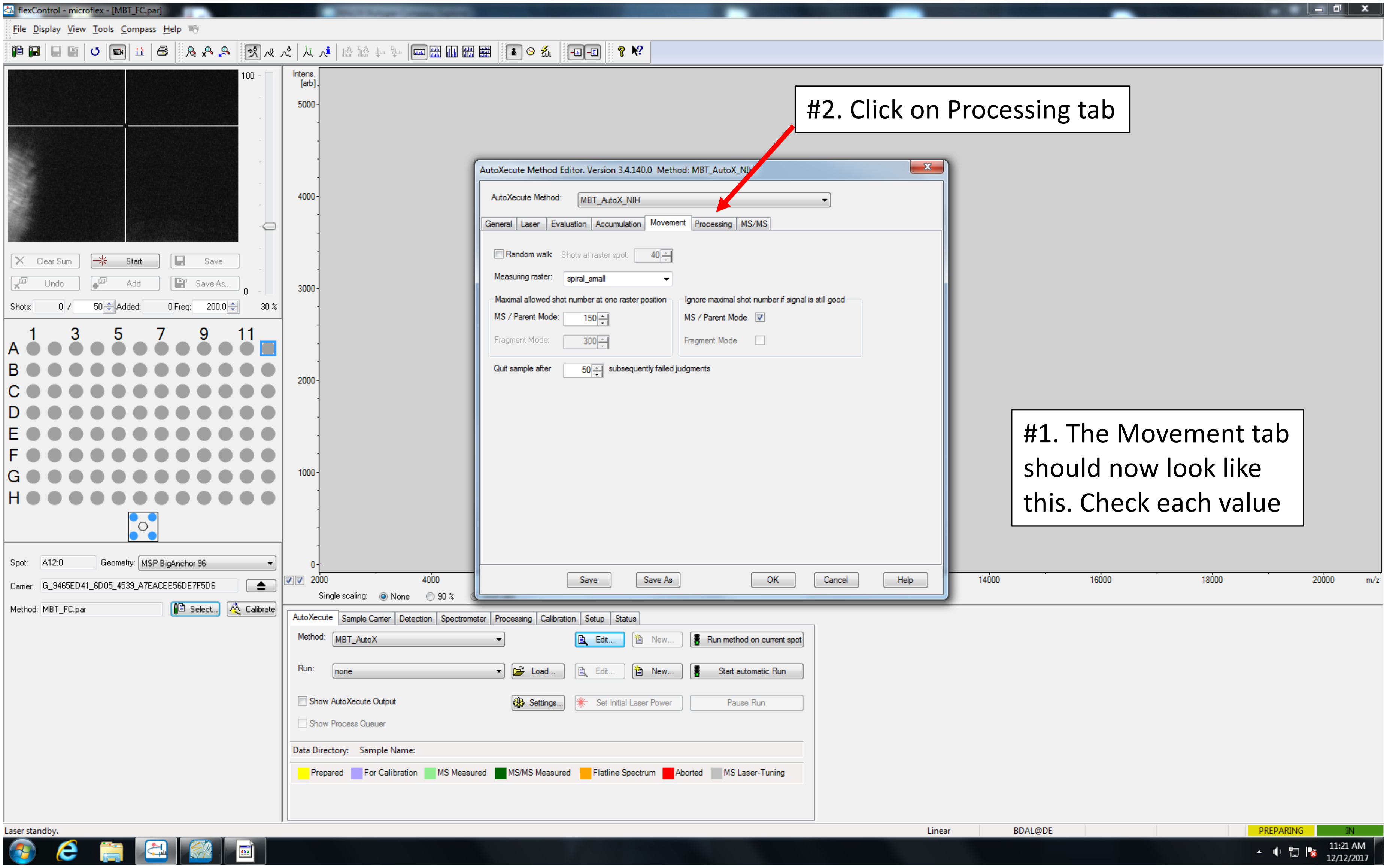

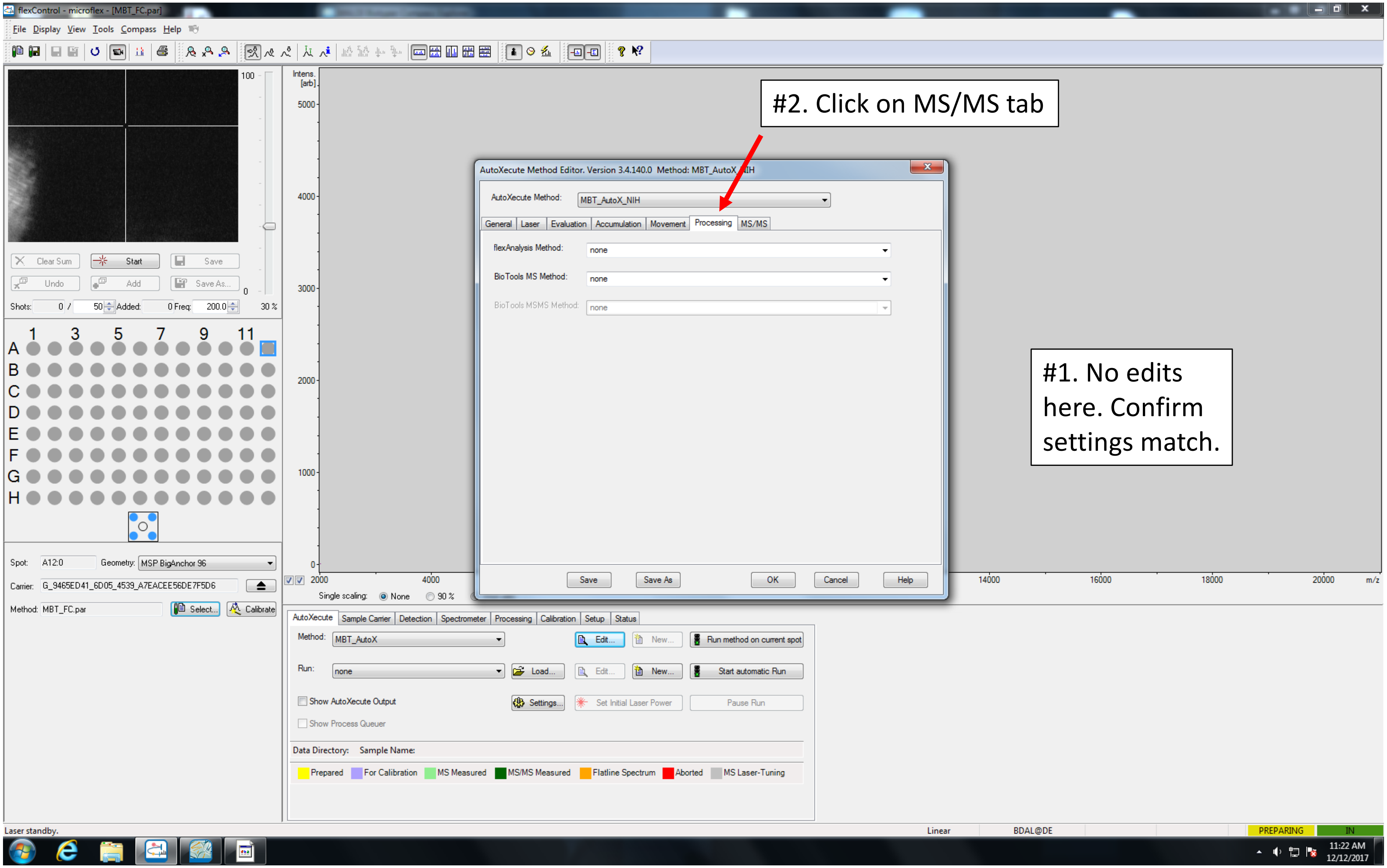
#2. Click on MS/MS tab
#1. No edits here. Confirm settings match.
flexControl - microflex - [MBT_FC.par]
File Display View Tools Compass Help
AutoXecute Method Editor. Version 3.4.140.0 Method: MBT_AutoX_NIH
AutoXecute Method:
MBT_AutoX_NIH
General
Laser
Evaluation
Accumulation
Movement
Processing
MS/MS
flexAnalysis Method:
none
BioTools MS Method:
none
BioTools MSMS Method:
none
Save
Save As
OK
Cancel
Help
Clear Sum
Start
Save
Undo
Add
Save As...
Spot:
A12:0
Geometry:
MSP BigAnchor 96
Carrier:
G_9465ED41_6D05_4539_A7EACEE56DE7F5D6
Method:
MBT_FC.par
Select...
Calibrate
AutoXecute
Sample Carrier
Detection
Spectrometer
Calibration
Setup
Status
Method:
MBT_AutoX
Edit...
New...
Run method on current spot
Run:
Load...
Start automatic Run
Show AutoXecute Output
Settings...
Set Initial Laser Power
Pause Run
Show Process Queuer
Data Directory:
Sample Name:
Prepared
For Calibration
MS Measured
MS/MS Measured
Flatline Spectrum
Aborted
MS Laser-Tuning
Laser standby.
Linear
BDAL@DE
PREPARING
IN
11:22 AM
12/12/2017

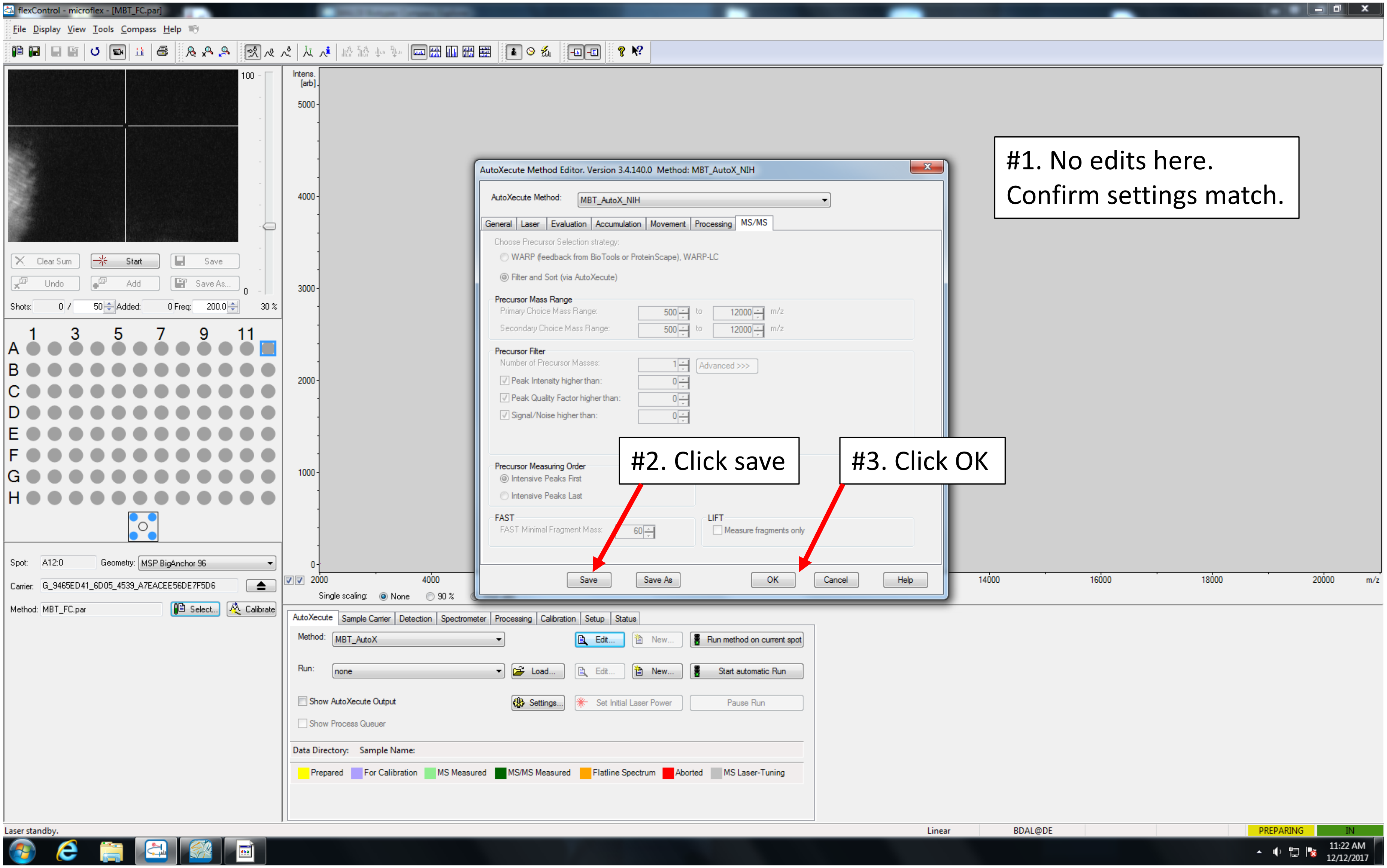
#1. No edits here.
Confirm settings match.
#2. Click save
#3. Click OK
AutoXecute Method Editor. Version 3.4.140.0 Method: MBT_AutoX_NIH
AutoXecute Method:
MBT_AutoX_NIH
General
Laser
Evaluation
Accumulation
Movement
Processing
MS/MS
Choose Precursor Selection strategy:
WARP (feedback from BioTools or ProteinScape), WARP-LC
Filter and Sort (via AutoXecute)
Precursor Mass Range
Primary Choice Mass Range:
Secondary Choice Mass Range:
Precursor Filter
Number of Precursor Masses:
Advanced >>>
Peak Intensity higher than:
Peak Quality Factor higher than:
Signal/Noise higher than:
Precursor Measuring Order
Intensive Peaks First
Intensive Peaks Last
FAST
FAST Minimal Fragment Mass:
LIFT
Measure fragments only
Save
Save As
OK
Cancel
Help
flexControl - microflex - [MBT_FC.par]
Method: MBT_FC.par
Geometry: MSP BigAnchor 96
Laser standby.

Embed Method into Compass

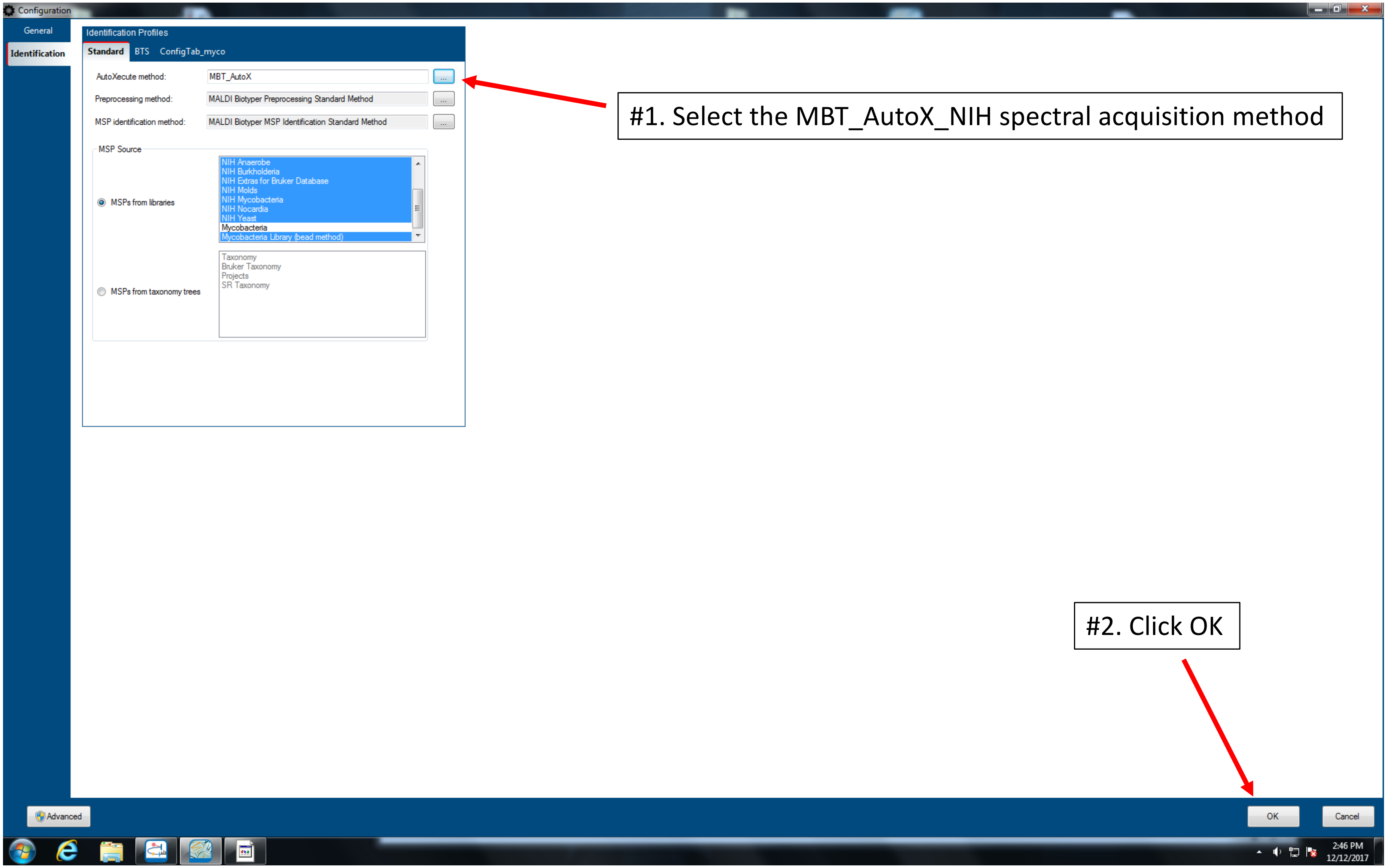
Configuration
General
Identification
Identification Profiles
Standard
BTS
ConfigTab_myco
AutoXecute method:
MBT_AutoX
Preprocessing method:
MALDI Biotyper Preprocessing Standard Method
MSP identification method:
MALDI Biotyper MSP Identification Standard Method
MSP Source
MSPs from libraries
NIH Anaerobe
NIH Burkholderia
NIH Extras for Bruker Database
NIH Molds
NIH Mycobacteria
NIH Nocardia
NIH Yeast
Mycobacteria
Mycobacteria Library (bead method)
MSPs from taxonomy trees
Taxonomy
Bruker Taxonomy
Projects
SR Taxonomy
#1. Select the MBT_AutoX_NIH spectral acquisition method
#2. Click OK
Advanced
OK
Cancel
2:46 PM
12/12/2017
