# Supplementary material for: Multicenter Study Demonstrates Standardization Requirements for Mold Identification by MALDI-TOF MS
Source: Front Microbiol. 2019 Sep 20;10:2098. doi: 10.3389/fmicb.2019.02098 (PMC6764242; doi:10.3389/fmicb.2019.02098)
Supplement: Supplementary file 2 [file Image_2.pdf]

Alternate-B
Spectral Acquisition Method

Supplemental Figure 2

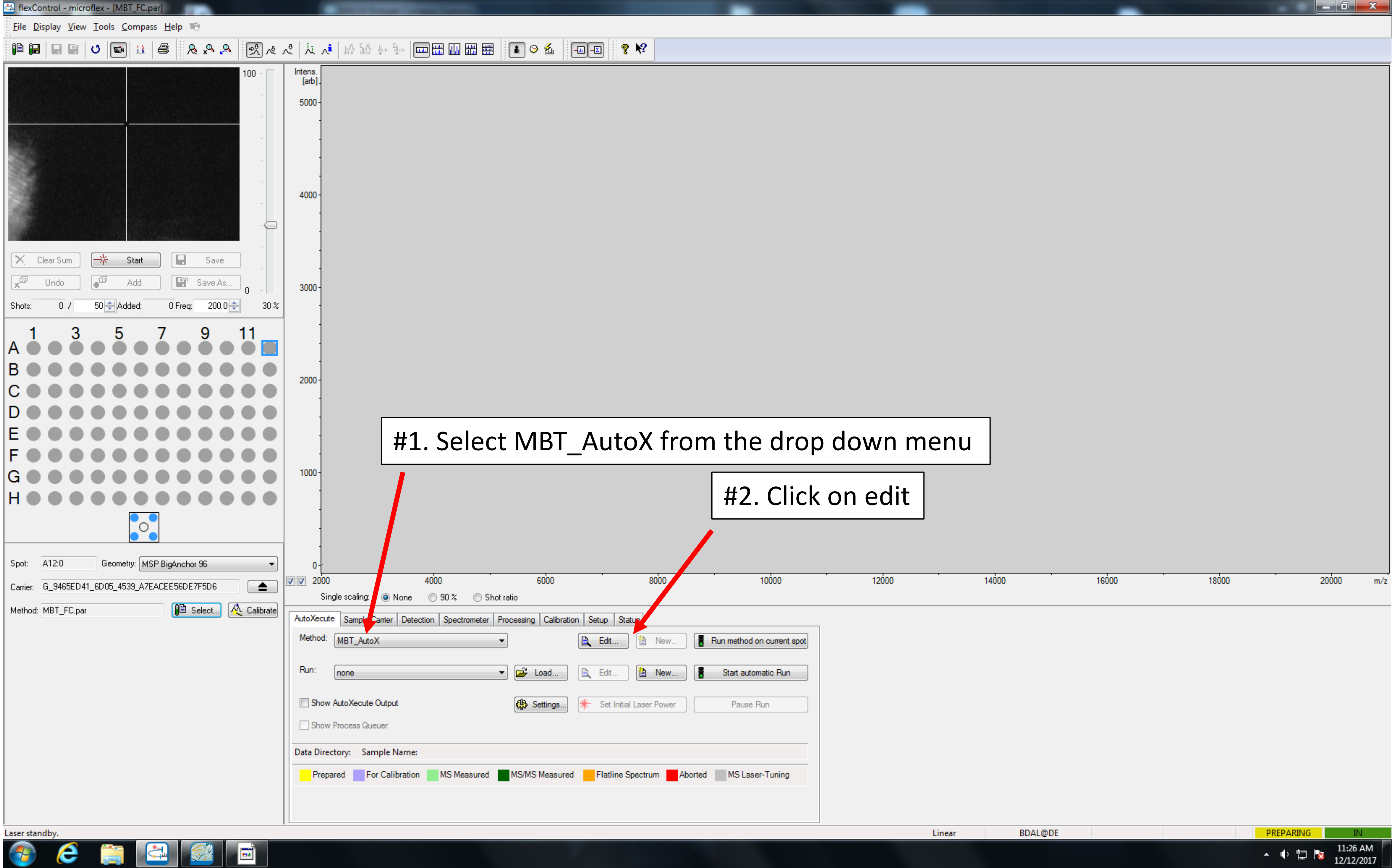
#1. Select MBT_AutoX from the drop down menu
#2. Click on edit
flexControl - microflex - [MBT_FC.par]
File Display View Tools Compass Help
Clear Sum
Start
Save
Undo
Add
Save As...
Shots: 0 / 50 Added: 0 Freq: 200.0 30 %
Spot: A12:0 Geometry: MSP BigAnchor 96
Carrier: G_9465ED41_6D05_4539_A7EACEE56DE7F5D6
Method: MBT_FC.par
Select...
Calibrate
Intens. [arb.]
m/z
Single scaling: None 90 % Shot ratio
AutoXecute Sample Carrier Detection Spectrometer Processing Calibration Setup Status
Method: MBT_AutoX
Edit...
New...
Run method on current spot
Run: none
Load...
Start automatic Run
Show AutoXecute Output
Settings...
Set Initial Laser Power
Pause Run
Show Process Queuer
Data Directory: Sample Name:
Prepared For Calibration MS Measured MS/MS Measured Flatline Spectrum Aborted MS Laser-Tuning
Laser standby.
Linear
BDAL@DE
PREPARING
IN
11:26 AM
12/12/2017

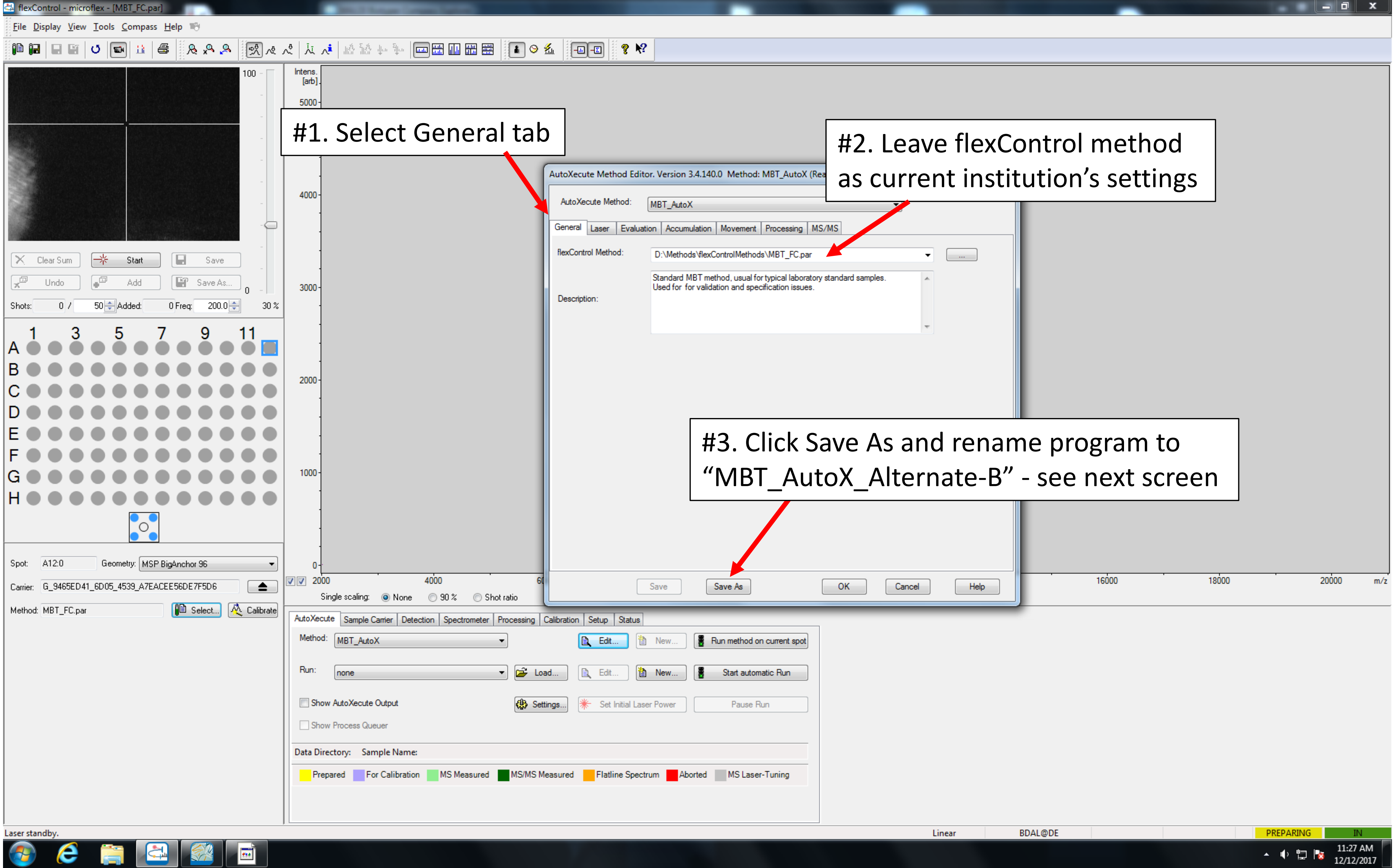
#1. Select General tab
#2. Leave flexControl method as current institution's settings
#3. Click Save As and rename program to "MBT_AutoX_Alternate-B" - see next screen
AutoXecute Method Editor. Version 3.4.140.0 Method: MBT_AutoX
AutoXecute Method: MBT_AutoX
General
Laser
Evaluation
Accumulation
Movement
Processing
MS/MS
flexControl Method: D:\Methods\flexControlMethods\MBT_FC.par
Description: Standard MBT method, usual for typical laboratory standard samples. Used for for validation and specification issues.
Save
Save As
OK
Cancel
Help

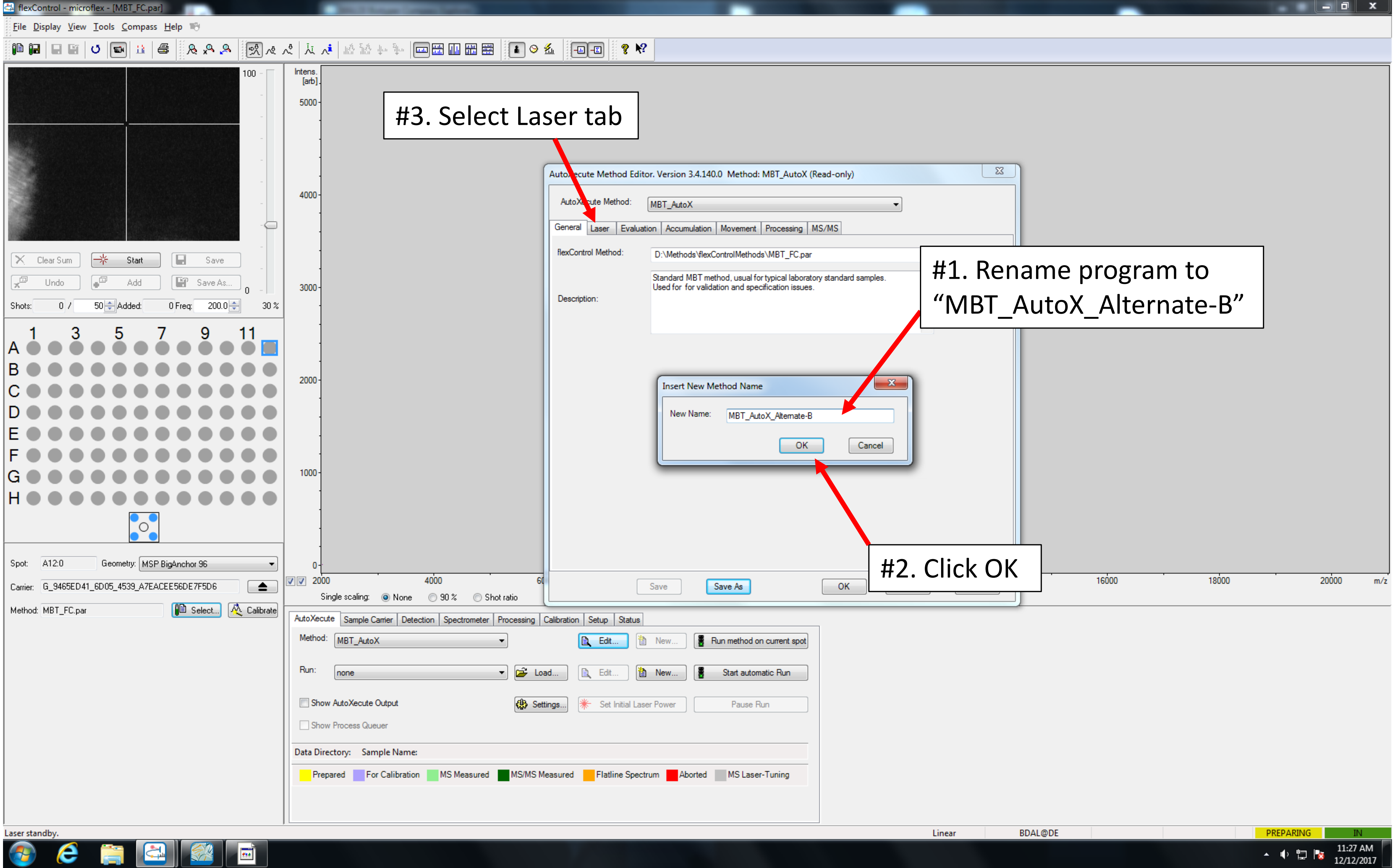

#3. Select Laser tab
#1. Rename program to "MBT_AutoX_Alternate-B"
#2. Click OK
AutoXecute Method Editor. Version 3.4.140.0 Method: MBT_AutoX (Read-only)
AutoXecute Method: MBT_AutoX
General
Laser
Evaluation
Accumulation
Movement
Processing
MS/MS
flexControl Method: D:\Methods\flexControlMethods\MBT_FC.par
Description: Standard MBT method, usual for typical laboratory standard samples. Used for for validation and specification issues.
Insert New Method Name
New Name: MBT_AutoX_Alternate-B
OK
Cancel
Save
Save As
OK

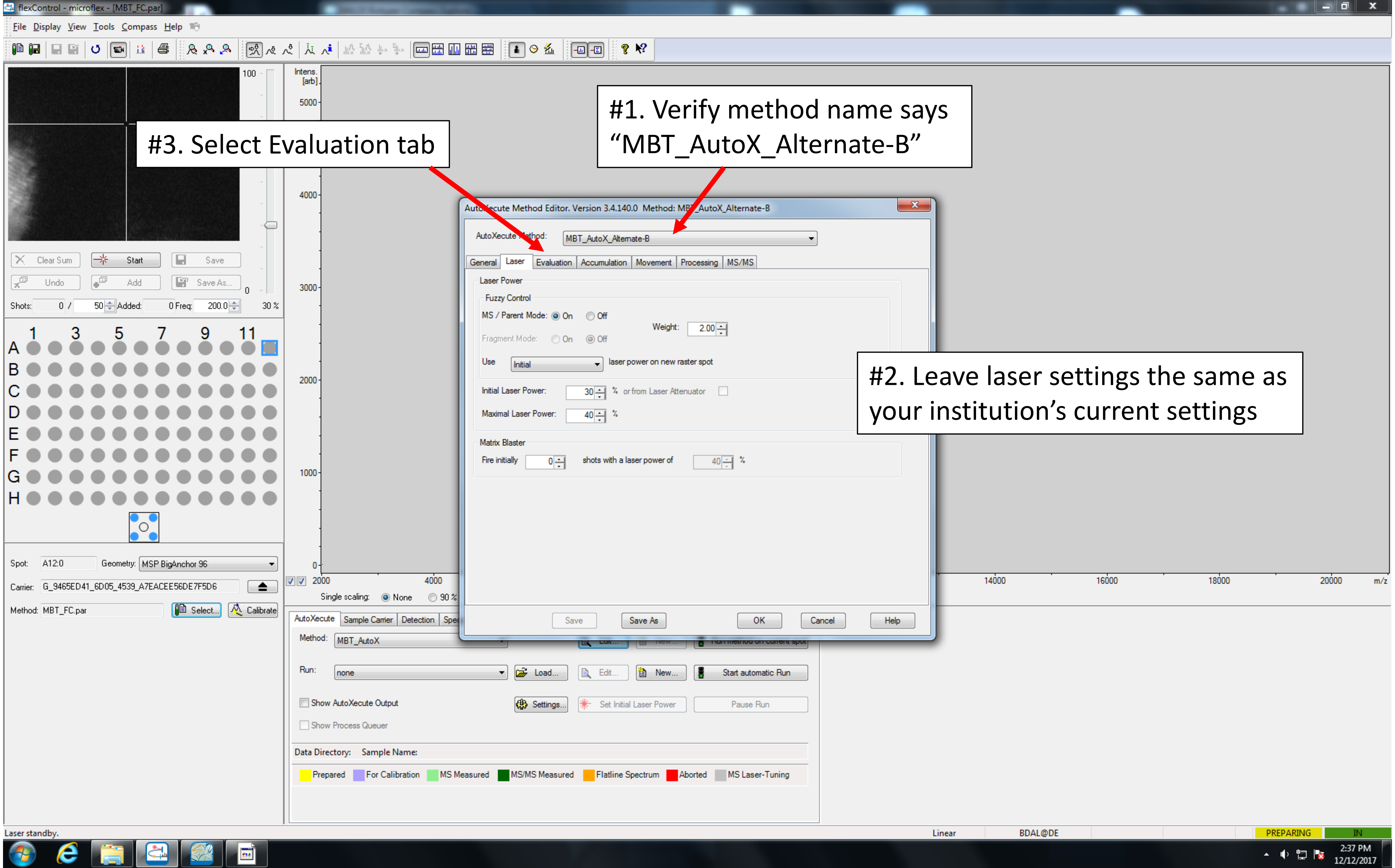
#1. Verify method name says “MBT_AutoX_Alternate-B”
#2. Leave laser settings the same as your institution’s current settings
#3. Select Evaluation tab
flexControl - microflex - [MBT_FC.par]
AutoXecute Method Editor. Version 3.4.140.0 Method: MBT_AutoX_Alternate-B
AutoXecute Method: MBT_AutoX_Alternate-B
General
Laser
Evaluation
Accumulation
Movement
Processing
MS/MS
Laser Power
Fuzzy Control
MS / Parent Mode: On Off
Weight: 2.00
Fragment Mode: On Off
Use Initial laser power on new raster spot
Initial Laser Power: 30 % or from Laser Attenuator
Maximal Laser Power: 40 %
Matrix Blaster
Fire initially 0 shots with a laser power of 40 %
Save
Save As
OK
Cancel
Help

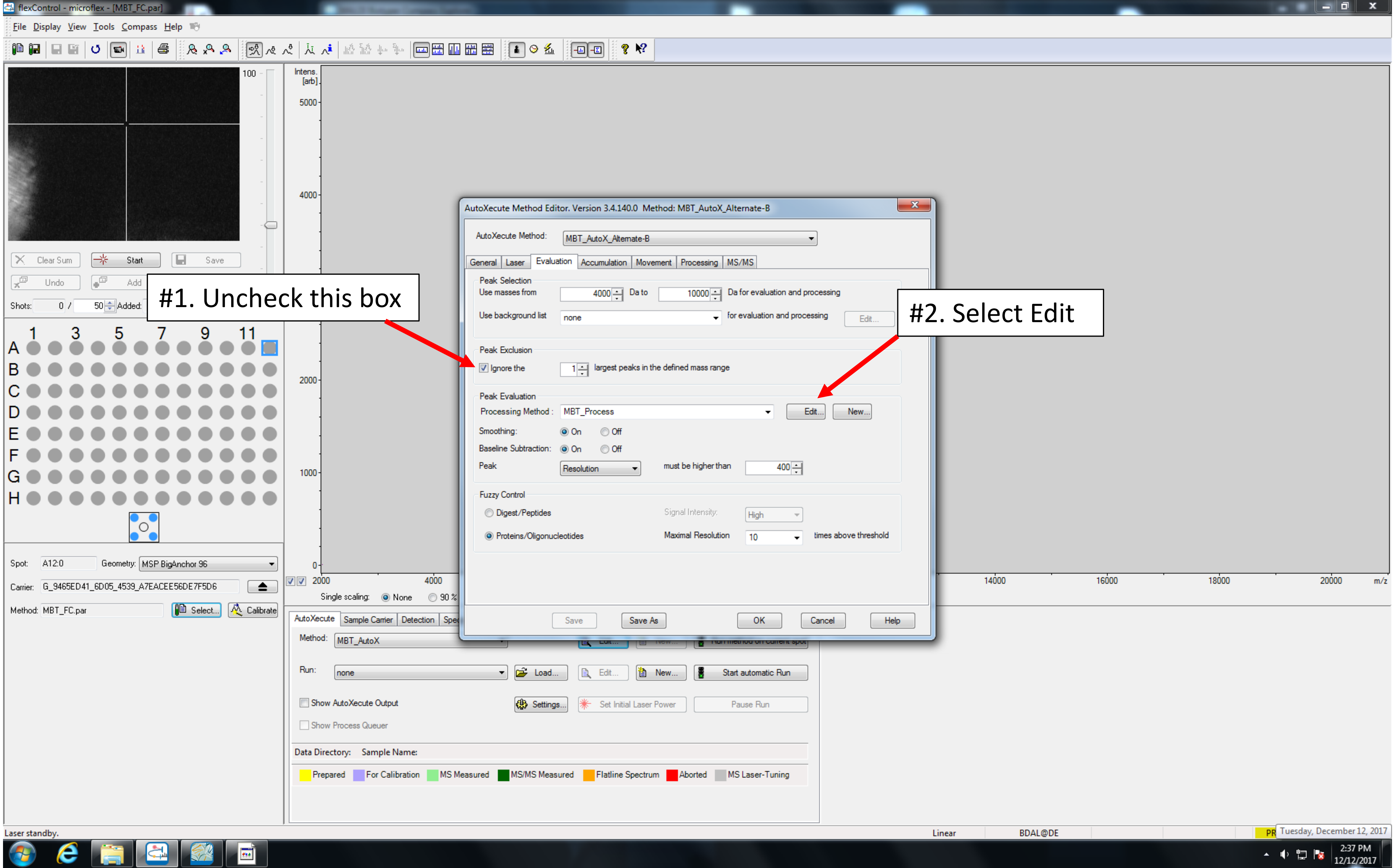
#1. Uncheck this box
#2. Select Edit
AutoXecute Method Editor. Version 3.4.140.0 Method: MBT_AutoX_Alternate-B
AutoXecute Method: MBT_AutoX_Alternate-B
General
Laser
Evaluation
Accumulation
Movement
Processing
MS/MS
Peak Selection
Use masses from 4000 Da to 10000 Da for evaluation and processing
Use background list none for evaluation and processing
Peak Exclusion
Ignore the 1 largest peaks in the defined mass range
Peak Evaluation
Processing Method : MBT_Process
Edit...
New...
Smoothing: On Off
Baseline Subtraction: On Off
Peak Resolution must be higher than 400
Fuzzy Control
Digest/Peptides
Signal Intensity: High
Proteins/Oligonucleotides
Maximal Resolution 10 times above threshold
Save
Save As
OK
Cancel
Help

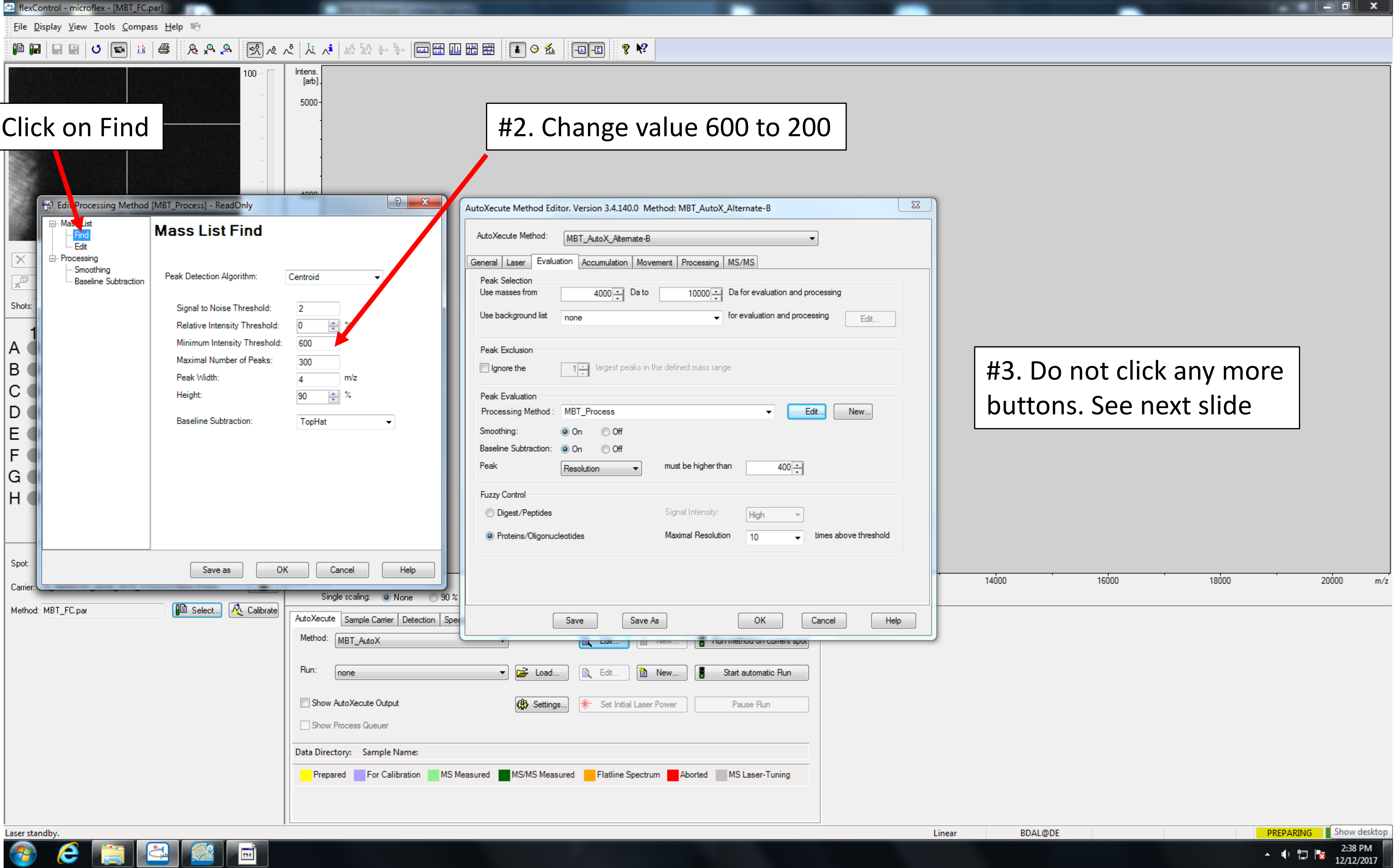
#1. Click on Find
#2. Change value 600 to 200
#3. Do not click any more buttons. See next slide
flexControl - microflex - [MBT_FC.par]
File Display View Tools Compass Help
Edit Processing Method [MBT_Process] - ReadOnly
Mass List
Find
Edit
Processing
Smoothing
Baseline Subtraction
Mass List Find
Peak Detection Algorithm: Centroid
Signal to Noise Threshold: 2
Relative Intensity Threshold: 0 %
Minimum Intensity Threshold: 600
Maximal Number of Peaks: 300
Peak Width: 4 m/z
Height: 90 %
Baseline Subtraction: TopHat
Save as
OK
Cancel
Help
AutoXecute Method Editor. Version 3.4.140.0 Method: MBT_AutoX_Alternate-B
AutoXecute Method: MBT_AutoX_Alternate-B
General Laser Evaluation Accumulation Movement Processing MS/MS
Peak Selection
Use masses from 4000 Da to 10000 Da for evaluation and processing
Use background list none for evaluation and processing
Peak Exclusion
Ignore the 1 largest peaks in the defined mass range
Peak Evaluation
Processing Method: MBT_Process
Smoothing: On Off
Baseline Subtraction: On Off
Peak Resolution must be higher than 400
Fuzzy Control
Digest/Peptides
Proteins/Oligonucleotides
Signal Intensity: High
Maximal Resolution 10 times above threshold
Save
Save As
OK
Cancel
Help
Method: MBT_FC.par
Select...
Calibrate
AutoXecute Sample Carrier Detection
Method: MBT_AutoX
Run: none
Load...
Edit...
New...
Start automatic Run
Show AutoXecute Output
Show Process Queuer
Settings...
Set Initial Laser Power
Pause Run
Data Directory: Sample Name:
Prepared For Calibration MS Measured MS/MS Measured Flatline Spectrum Aborted MS Laser-Tuning
Laser standby.
PREPARING

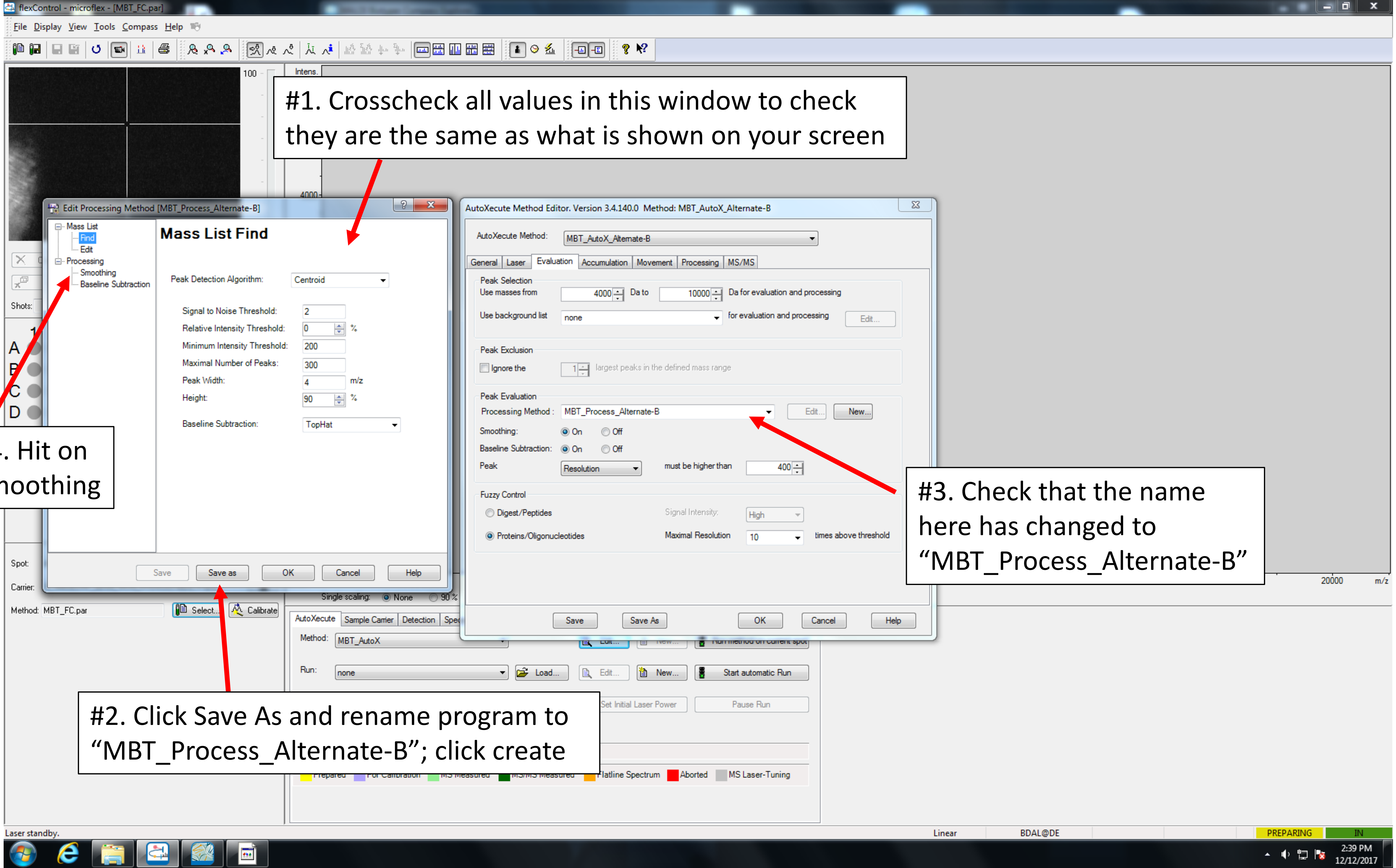

#1. Crosscheck all values in this window to check they are the same as what is shown on your screen

#2. Click Save As and rename program to “MBT_Process_Alternate-B”; click create

#3. Check that the name here has changed to “MBT_Process_Alternate-B”

#4. Hit on Smoothing

#1. No edits on this screen. Confirm that values match what is shown here

#2. Click on Baseline subtraction

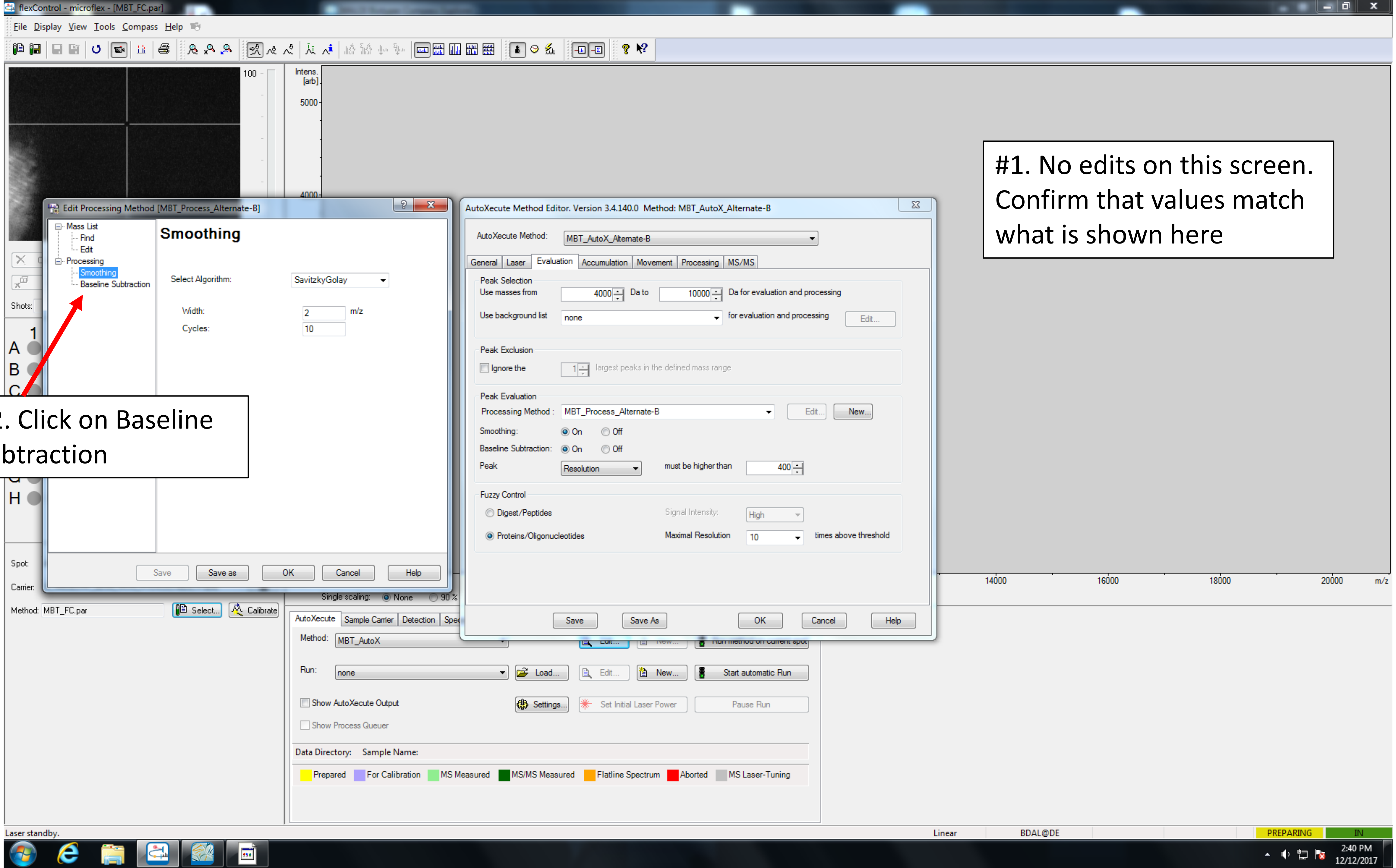

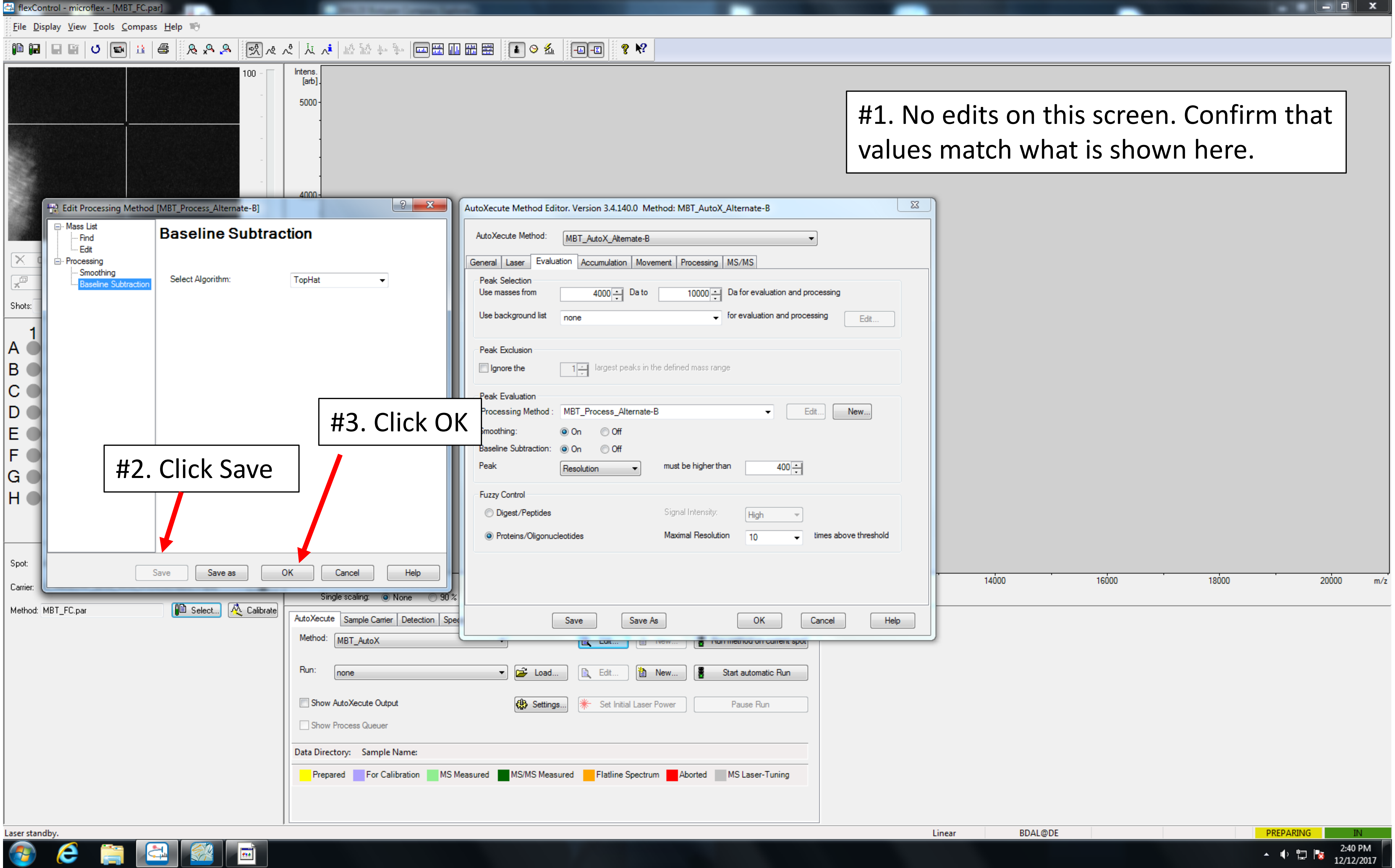
#1. No edits on this screen. Confirm that values match what is shown here.
#2. Click Save
#3. Click OK
Edit Processing Method [MBT_Process_Alternate-B]
Baseline Subtraction
Select Algorithm: TopHat
Save
Save as
OK
Cancel
Help
AutoXecute Method Editor. Version 3.4.140.0 Method: MBT_AutoX_Alternate-B
AutoXecute Method: MBT_AutoX_Alternate-B
General
Laser
Evaluation
Accumulation
Movement
Processing
MS/MS
Peak Selection
Use masses from 4000 Da to 10000 Da for evaluation and processing
Use background list none for evaluation and processing
Peak Exclusion
Ignore the 1 largest peaks in the defined mass range
Peak Evaluation
Processing Method: MBT_Process_Alternate-B
Smoothing: On Off
Baseline Subtraction: On Off
Peak Resolution must be higher than 400
Fuzzy Control
Digest/Peptides
Proteins/Oligonucleotides
Signal Intensity: High
Maximal Resolution 10 times above threshold
Save
Save As
OK
Cancel
Help

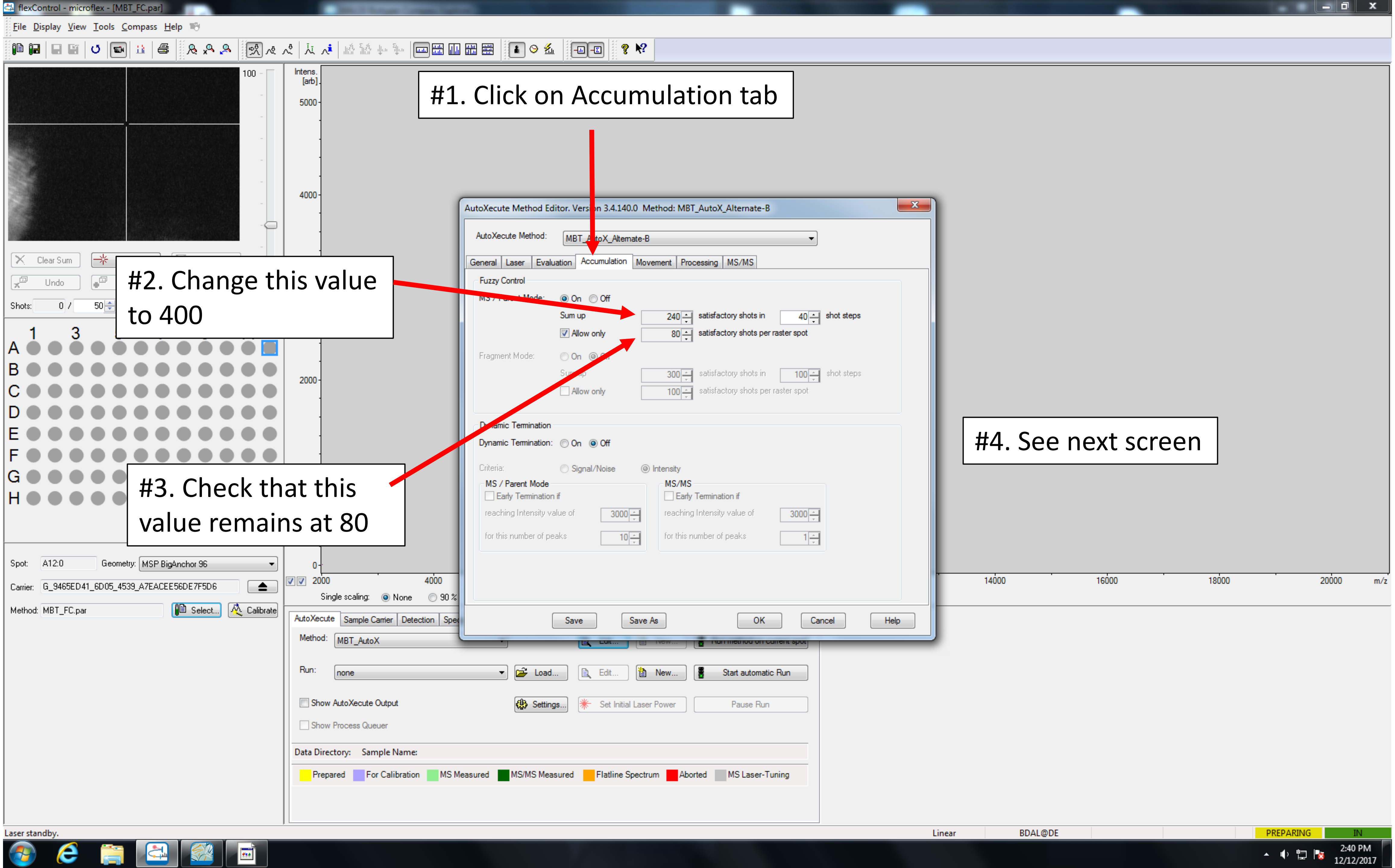
#1. Click on Accumulation tab
#2. Change this value to 400
#3. Check that this value remains at 80
#4. See next screen
AutoXecute Method Editor. Version 3.4.140.0 Method: MBT_AutoX_Alternate-B
AutoXecute Method: MBT_AutoX_Alternate-B
General
Laser
Evaluation
Accumulation
Movement
Processing
MS/MS
Fuzzy Control
Sum up
240 satisfactory shots in 40 shot steps
Allow only
80 satisfactory shots per raster spot
Fragment Mode:
300 satisfactory shots in 100 shot steps
100 satisfactory shots per raster spot
Dynamic Termination: On Off
Criteria: Signal/Noise Intensity
MS / Parent Mode
MS/MS
Early Termination if
reaching Intensity value of 3000
for this number of peaks
Save
Save As
OK
Cancel
Help
flexControl - microflex - [MBT_FC.par]
Laser standby.
PREPARING
2:40 PM
12/12/2017

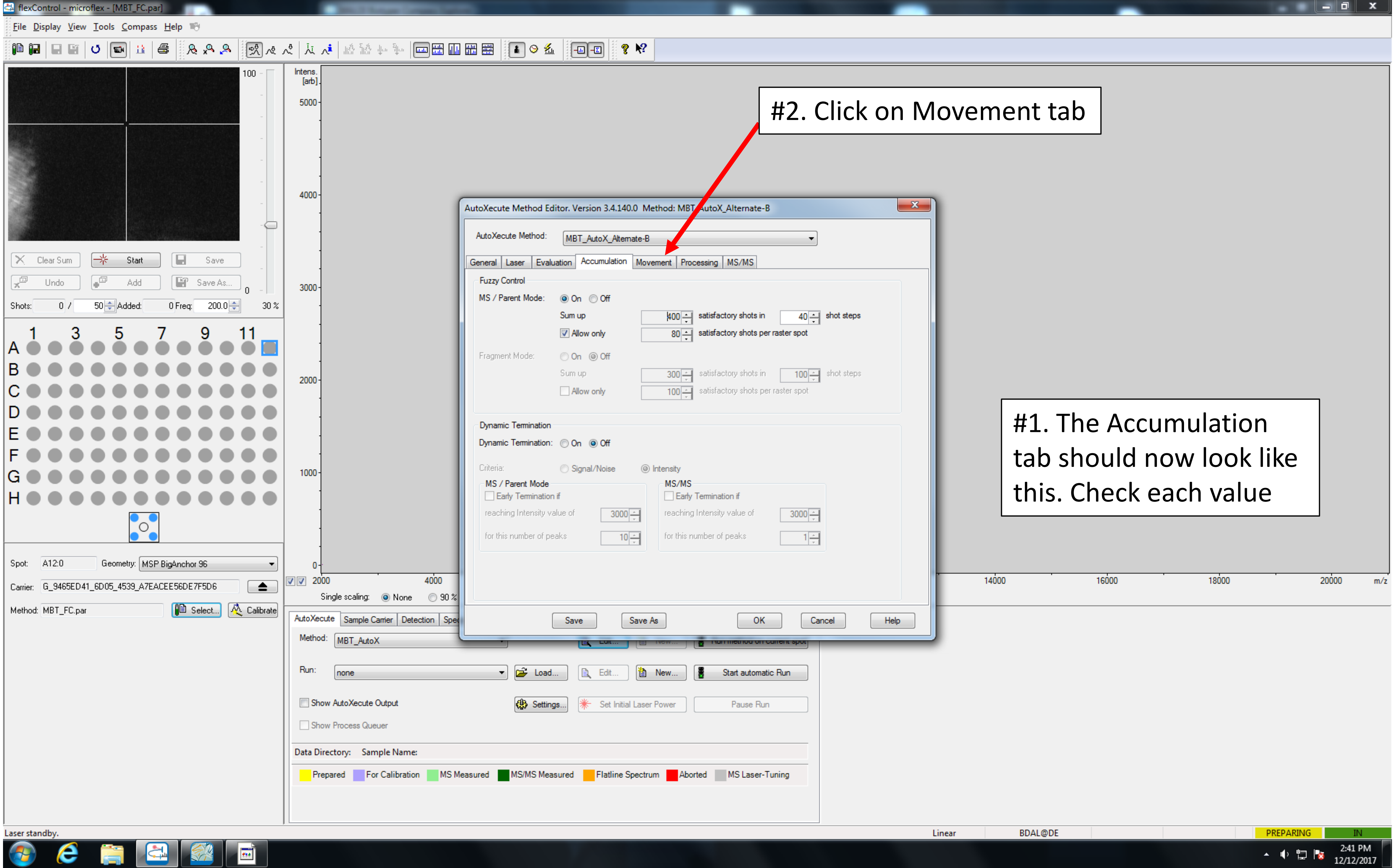
#2. Click on Movement tab
#1. The Accumulation tab should now look like this. Check each value
AutoXecute Method Editor. Version 3.4.140.0 Method: MBT_AutoX_Alternate-B
AutoXecute Method: MBT_AutoX_Alternate-B
General
Laser
Evaluation
Accumulation
Movement
Processing
MS/MS
Fuzzy Control
MS / Parent Mode: On Off
Sum up 400 satisfactory shots in 40 shot steps
Allow only 80 satisfactory shots per raster spot
Fragment Mode: On Off
Sum up 300 satisfactory shots in 100 shot steps
Allow only 100 satisfactory shots per raster spot
Dynamic Termination
Dynamic Termination: On Off
Criteria: Signal/Noise Intensity
MS / Parent Mode
Early Termination if
reaching Intensity value of 3000
for this number of peaks 10
MS/MS
Early Termination if
reaching Intensity value of 3000
for this number of peaks 1
Save
Save As
OK
Cancel
Help

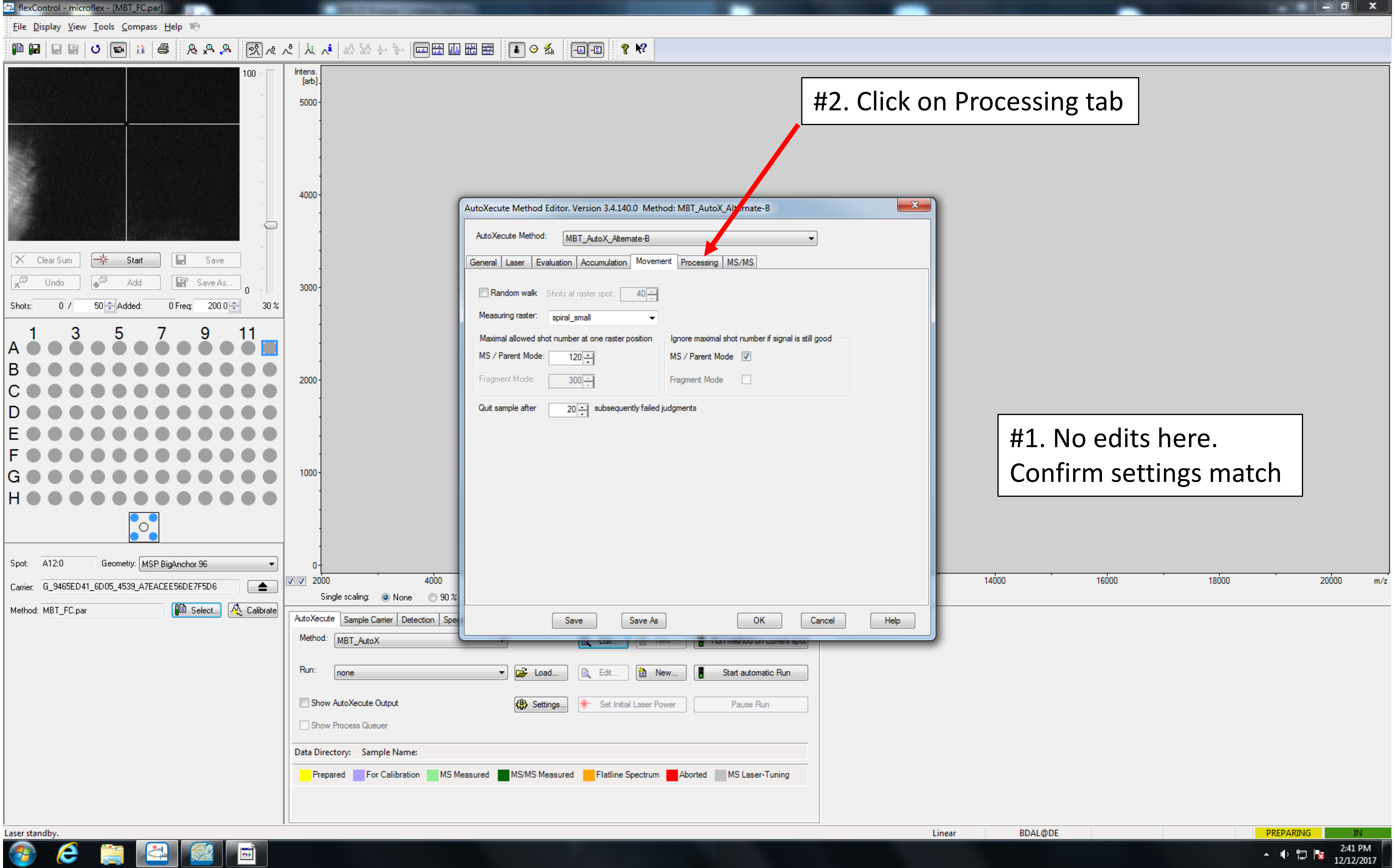
#2. Click on Processing tab
#1. No edits here. Confirm settings match
flexControl - microflex - [MBT_FC.par]
AutoXecute Method Editor. Version 3.4.140.0 Method: MBT_AutoX_Alternate-B
AutoXecute Method: MBT_AutoX_Alternate-B
General Laser Evaluation Accumulation Movement Processing MS/MS
Random walk
Shots at raster spot: 40
Measuring raster: spiral_small
Maximal allowed shot number at one raster position
MS / Parent Mode: 120
Fragment Mode: 300
Ignore maximal shot number if signal is still good
MS / Parent Mode
Fragment Mode
Quit sample after 20 subsequently failed judgments
Save Save As OK Cancel Help
Spot: A12:0
Geometry: MSP BigAnchor 96
Carrier: G_9465ED41_6D05_4539_A7EACEE56DE7F5D6
Method: MBT_FC.par
Method: MBT_AutoX
Run: none
Start automatic Run
Show AutoXecute Output
Show Process Queuer
Prepared For Calibration MS Measured MS/MS Measured Flatline Spectrum Aborted MS Laser-Tuning
Laser standby.
PREPARING

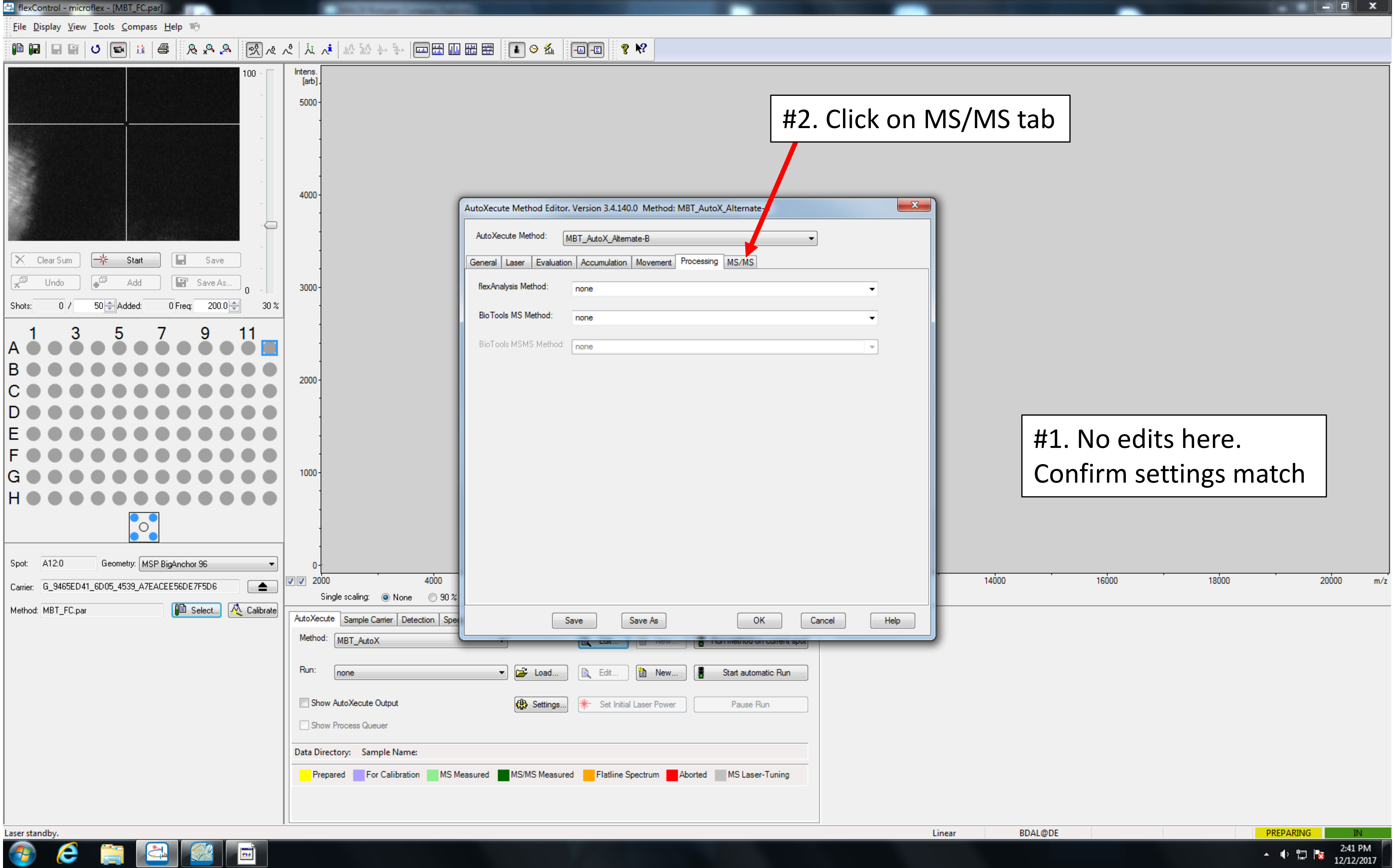

#2. Click on MS/MS tab

#1. No edits here. Confirm settings match

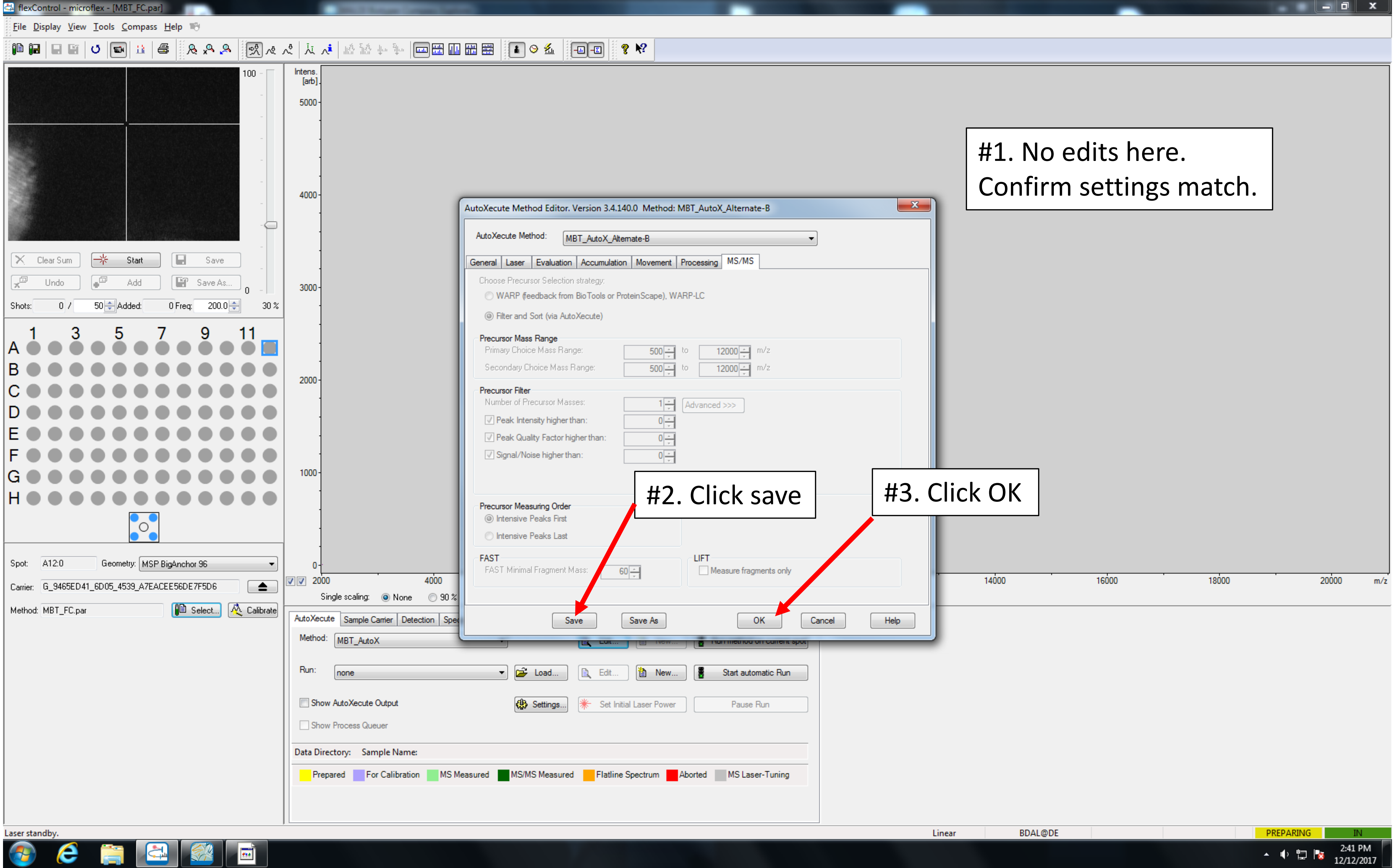
#1. No edits here.
Confirm settings match.
#2. Click save
#3. Click OK
flexControl - microflex - [MBT_FC.par]
File Display View Tools Compass Help
AutoXecute Method Editor. Version 3.4.140.0 Method: MBT_AutoX_Alternate-B
AutoXecute Method: MBT_AutoX_Alternate-B
General Laser Evaluation Accumulation Movement Processing MS/MS
Choose Precursor Selection strategy:
WARP (feedback from BioTools or ProteinScape), WARP-LC
Filter and Sort (via AutoXecute)
Precursor Mass Range
Primary Choice Mass Range: 500 to 12000 m/z
Secondary Choice Mass Range: 500 to 12000 m/z
Precursor Filter
Number of Precursor Masses: 1 Advanced >>>
Peak Intensity higher than: 0
Peak Quality Factor higher than: 0
Signal/Noise higher than: 0
Precursor Measuring Order
Intensive Peaks First
Intensive Peaks Last
FAST
FAST Minimal Fragment Mass: 60
LIFT
Measure fragments only
Save Save As OK Cancel Help
Clear Sum Start Save
Undo Add Save As...
Shots: 0 / 50 Added: 0 Freq: 200.0 30 %
Spot: A12:0 Geometry: MSP BigAnchor 96
Carrier: G_9465ED41_6D05_4539_A7EACEE56DE7F5D6
Method: MBT_FC.par Select... Calibrate
AutoXecute Sample Carrier Detection
Method: MBT_AutoX
Run: none Load... Edit... New... Start automatic Run
Show AutoXecute Output Settings... Set Initial Laser Power Pause Run
Show Process Queuer
Data Directory: Sample Name:
Prepared For Calibration MS Measured MS/MS Measured Flatline Spectrum Aborted MS Laser-Tuning
Laser standby.
Linear BDAL@DE PREPARING IN
2:41 PM 12/12/2017

Embed Method into Compass

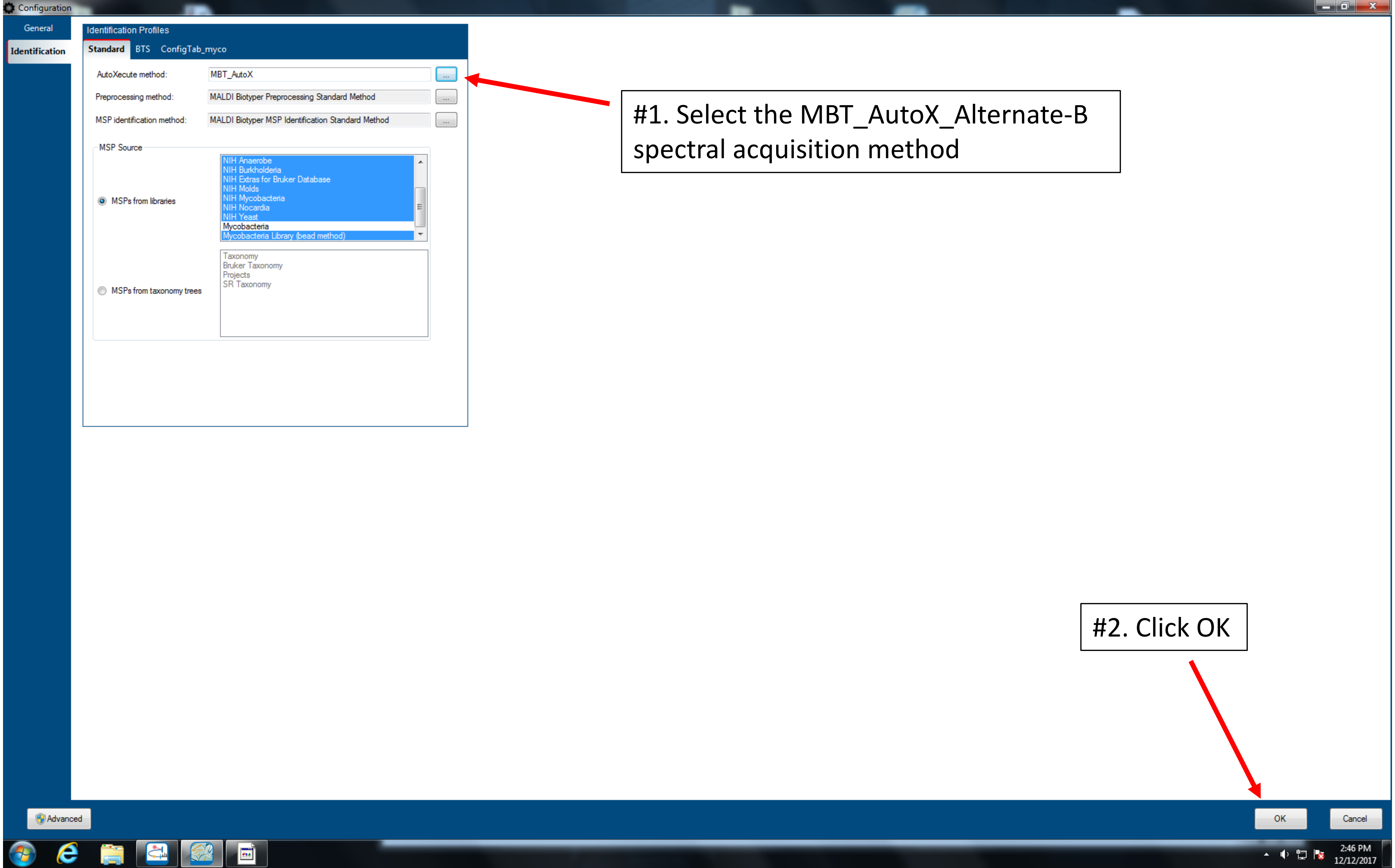

#1. Select the MBT_AutoX_Alternate-B spectral acquisition method

#2. Click OK
