# Supplementary material for: Multicenter Study Demonstrates Standardization Requirements for Mold Identification by MALDI-TOF MS
Source: Front Microbiol. 2019 Sep 20;10:2098. doi: 10.3389/fmicb.2019.02098 (PMC6764242; doi:10.3389/fmicb.2019.02098)
Supplement: Supplementary file 3 [file Image_3.pdf]

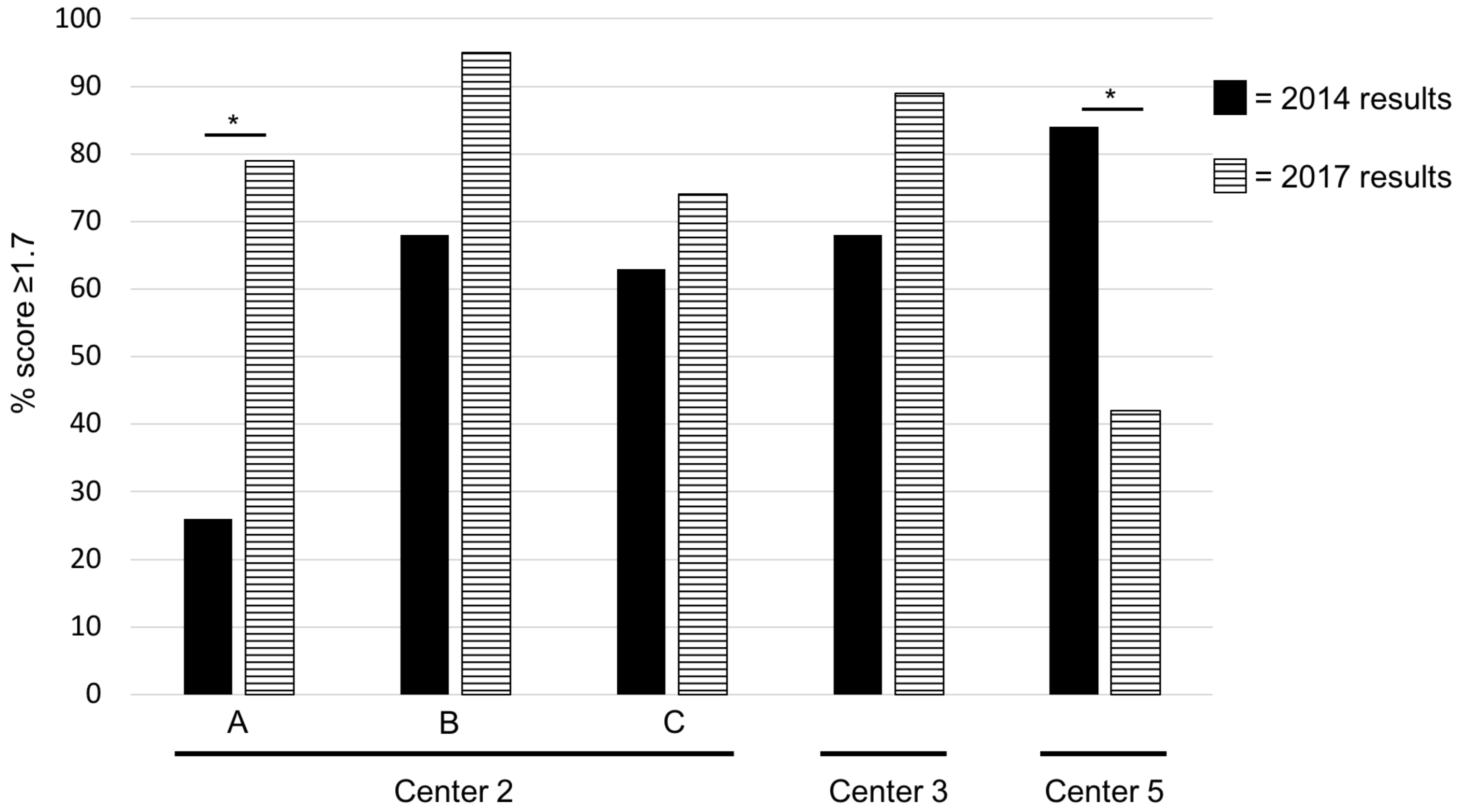

Supplemental Figure 3. Comparative Performance of MBT-AutoX Method During Initial Analysis (2014) with Later Analysis (2017) Across Multiple Institutions. Paired isolates (n=17; marked by * in Table 1) from original results after watching the video in 2014 (black bars) versus performance in 2017 (lined bars) on the same instruments using fresh extracts, NIH extraction method, MBT_AutoX acquisition method, and NIH database. Instruments A, B, and C represent three different instruments at Center 2. *denotes p-value <0.05.
