# Supplementary material for: Multicenter Study Demonstrates Standardization Requirements for Mold Identification by MALDI-TOF MS
Source: Front Microbiol. 2019 Sep 20;10:2098. doi: 10.3389/fmicb.2019.02098 (PMC6764242; doi:10.3389/fmicb.2019.02098)
Supplement: Supplementary file 4 [file Image_4.pdf]

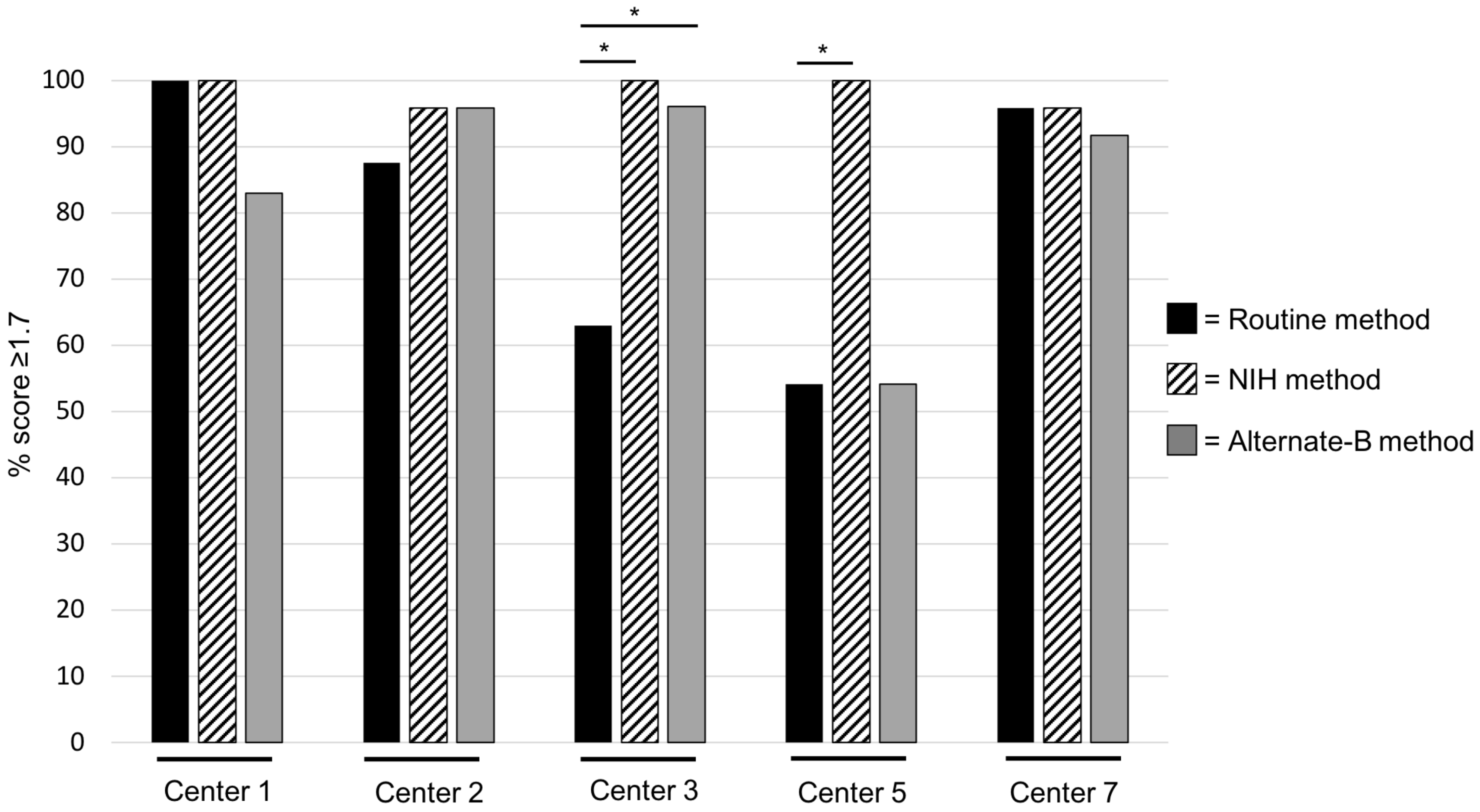

Supplemental Figure 4. Pilot Comparative Analysis of Three Different Spectral Acquisition Methods Using a Single Prepared Plate. Fresh extracts for six isolates were distributed to each institution and spotted in quadruplicate onto a single plate (n=24). Spectra were acquired on the same day in this order of spectral acquisition methods: 1) routine spectral acquisition method at that institution (NIH method at Center 1, MBT_AutoX method at Centers 2, 3, 5, and 7; black bars); 2) NIH method (hashed bars); and, 3) Alternate-B method (grey bars). *denotes p-value <0.05.
