# Supplementary material for: Multicenter Study Demonstrates Standardization Requirements for Mold Identification by MALDI-TOF MS
Source: Front Microbiol. 2019 Sep 20;10:2098. doi: 10.3389/fmicb.2019.02098 (PMC6764242; doi:10.3389/fmicb.2019.02098)
Supplement: Supplementary file 5 [file Table_1.pdf]

Supplementary Table 1. Pilot study using 17 isolates (marked by * in Table 1) extracted at the NIH at Centers 1 and 2 evaluating pre- and post- instrument optimization using routine spectral acquisition methods for each site, and performance on the instrument at Bruker US headquarters at Billerica, MA.

**Description**	**Acquisition method**	**Log score ≥1.7 (%)**	**p-value**
Center 1 (NIH) pre-optimization	NIH method	76	0.3
Center 1 (NIH) post-optimization	NIH method	94	
Center 2 pre-optimization	MBT_AutoX	59	0.0002
Center 2 post-optimization	MBT_AutoX	31	0.2; 1.0; 1.0
Center 2 extracts returned to NIH	NIH method	88	0.001; <0.0001
Bruker US Headquarters	MBT_AutoX	100	
