# Supplementary material for: Multicenter Study Demonstrates Standardization Requirements for Mold Identification by MALDI-TOF MS
Source: Front Microbiol. 2019 Sep 20;10:2098. doi: 10.3389/fmicb.2019.02098 (PMC6764242; doi:10.3389/fmicb.2019.02098)
Supplement: Supplementary file 6 [file Table_2.pdf]

Supplementary Table 2. Setting differences identified between three spectral acquisition parameters

**Parameter**	**MBT_AutoX**	**NIH method**	**Alternate-B method**
Peak selection	4000-10000	2000-20000	4000-10000
Minimum intensity threshold	600	30	200
Sum shots acquired	240	250	400
